# Supramolecular associations between atypical oxidative phosphorylation complexes of *Euglena gracilis*

**DOI:** 10.1007/s10863-021-09882-8

**Published:** 2021-03-01

**Authors:** H. V. Miranda-Astudillo, K. N. S. Yadav, E. J. Boekema, P. Cardol

**Affiliations:** 1 InBios/Phytosystems, Institut de Botanique, University of Liège, Liège, Belgium; 2 Present Address: Departamento de Biología Molecular y Biotecnología, Instituto de Investigaciones Biomédicas, Universidad Nacional Autónoma de México, Mexico City, Mexico; 3 Department of Electron Microscopy, Groningen Biological Sciences and Biotechnology Institute, University of Groningen, Groningen, the Netherlands; 4 Present Address: School of Biochemistry, University of Bristol, Bristol, BS8 1TD UK

**Keywords:** *Euglena gracilis*, Oxidative phosphorylation, F_1_F_O_ ATP synthase, Oligomeric complex V, Mitochondrial supercomplexes, Respirasome

## Abstract

In vivo associations of respiratory complexes forming higher supramolecular structures are generally accepted nowadays. Supercomplexes (SC) built by complexes I, III and IV and the so-called respirasome (I/III_2_/IV) have been described in mitochondria from several model organisms (yeasts, mammals and green plants), but information is scarce in other lineages. Here we studied the supramolecular associations between the complexes I, III, IV and V from the secondary photosynthetic flagellate *Euglena gracilis* with an approach that involves the extraction with several mild detergents followed by native electrophoresis. Despite the presence of atypical subunit composition and additional structural domains described in *Euglena* complexes I, IV and V, canonical associations into III_2_/IV, III_2_/IV_2_ SCs and I/III_2_/IV respirasome were observed together with two oligomeric forms of the ATP synthase (V_2_ and V_4_). Among them, III_2_/IV SC could be observed by electron microscopy. The respirasome was further purified by two-step liquid chromatography and showed in-vitro oxygen consumption independent of the addition of external cytochrome *c*.

**Supplementary Information:**

The online version contains supplementary material available at 10.1007/s10863-021-09882-8.

## Introduction

ATP production by oxidative phosphorylation (OXPHOS) is a key process in eukaryotic energetic metabolism. In this process, the respiratory chain complexes NADH:ubiquinone oxidoreductase (complex I), succinate:ubiquinone oxidoreductase (complex II), ubiquinol:cytochrome *c* oxidoreductase (complex III) and cytochrome *c* oxidase (complex IV) transfer electrons from NADH or succinate to oxygen and, except for complex II, establish an electrochemical proton gradient across the inner mitochondrial membrane (proton-motive force). Two mobile electron carriers, ubiquinone and cytochrome *c*, connect the electron flow between complex I or II with complex III, and complex III with IV, respectively. An additional complex, the ATP synthase (complex V), utilizes the energy of the proton-motive force to synthetize ATP.

The organization of the OXPHOS complexes is generally discussed in terms of two extreme models, the “fluid state” where all membrane proteins and redox components are in constant and independent diffusional motion (Hackenbrock et al. 1986) in agreement with the “fluid mosaic model” (Singer and Nicolson 1972) and the “solid state” model which proposes that all the complexes are associated in one functional unit (Keilin and Hartree 1947; Slater 2003). The first suggestion that the OXPHOS complexes can associate with each other in larger structures named supercomplexes (SC) was brought to light based on pioneering blue native electrophoresis experiments (Schägger and Pfeiffer 2000). With the advance of techniques to obtain larger macromolecular protein structures, e.g. cryo-electron microscopy and cryotomography, nowadays, the existence of mitochondrial SC is generally accepted (Wittig and Schägger 2009; Chaban et al. 2014; Genova and Lenaz 2014; Letts et al. 2016; Lobo-Jarne and Ugalde 2017). The reasons or advantages of these association are still in debate, they include a more efficient transport of electrons to minimize the generation of reactive oxygen species during electron transfer reactions (Winge 2012), the regulation of the mitochondrial metabolism in response to different stimuli, carbon sources, or stress conditions (Acin-Perez and Enriquez 2014; Genova and Lenaz 2014) or provide a kinetic advantage by substrate channeling specially maintaining a dedicated quinol pool (Genova and Lenaz 2014; Lenaz et al. 2016; Milenkovic et al. 2017).

The family of the rotary ATPases originates from a common evolutionary ancestor (Cross and Taiz 1990; Cross and Müller 2004). This superfamily comprises the vacuolar H^+^-translocating V_1_V_o_-ATPases (V-ATPase), the archaeal A_1_A_o_-ATPases (A-ATPase) and the bacterial, plastid and mitochondrial F_1_F_o_-ATPases (F-ATPase) (Muench et al. 2011). The overall structure and subunit composition of the bacterial and mitochondrial F-ATPases from Opisthokonts (i.e. Fungi/Metazoa group), is overall well conserved (Kühlbrandt 2019). The mitochondrial F-ATPase presents a dimeric nature with a V-shape architecture that folds the inner membrane to form the mitochondrial cristae (Strauss et al. 2008; Davies et al. 2011). In contrast, no bacterial or plastidic dimeric enzyme has been reported to date. Accordingly, the most variable structure among the different species is the peripheral stalk while the most conserved regions between bacterial and opisthokont enzymes correspond to the catalytic core (α_3_β_3_), the peripheral stalk binding subunit (OSCP), the central rotor and proton-translocation region (γ, δ, ε, *a* and *c*-ring) (Colina-Tenorio et al. 2018). The study of mitochondrial F-ATPase of other eukaryotic lineages (e.g. ciliates (Alveolata), chlorophyceae (Archeaeplastida), euglenozoan (Excavata)) revealed highly divergent subunit compositions of the peripheral stalk between the lineages (Zíková et al. 2009; Balabaskaran Nina et al. 2010; Allegretti et al. 2015; Mühleip et al. 2016, 2017; Yadav et al. 2017; Miranda-Astudillo et al. 2018a; Salunke et al. 2018; Colina-Tenorio et al. 2019). These various peripheral stalks are usually more robust, and give rise to highly stable dimers with various geometries.

*Euglena gracilis* is a secondary photosynthetic flagellate that arose from an endosymbiosis between a green alga and an ancient phagotroph (Gibbs 1981; Turmel et al. 2009). Euglenids, together with other heterotrophic flagellates like Symbiontida (free-living flagellates found in low-oxygen marine sediments), Diplonemea (free-living marine flagellates) and Kinetoplastida (free-living and parasitic flagellates, e.g. *Trypanosoma*) form the monophyletic Euglenozoa group (Burki 2014; Zakrys et al. 2017). *E. gracilis* has a mitochondrial electron transfer system constituted by the OXPHOS complexes (Complexes I - IV) and also exhibits alternative electron pathways. These pathways involve an alternative oxidase (AOX) sensitive to diphenylamine, salicylhydroxamic acid (SHAM), n-propyl gallate and disulfiram (Sharpless and Butow 1970a; Benichou et al. 1988; Moreno-Sánchez et al. 2000), a CIII-like complex resistant to antimycin A (Sharpless and Butow 1970b) and an enzyme catalyzing a cytochrome *c* oxidase activity partially insensitive to cyanide in the presence of L-lactate (Moreno-Sánchez et al. 2000).

The subunit composition of the OXPHOS complexes among the Euglenozoa species includes the conserved canonical subunits, mainly related with the catalytic activity of each complex, but also a series of lineage-specific atypical subunits (Speijer et al. 1997; Morales et al. 2009; Perez et al. 2014; Verner et al. 2015; Yadav et al. 2017; Miranda-Astudillo et al. 2018b). This divergent subunit composition leads notably to the presence of atypical domains observed in the structures of complexes I, IV and V_2_ (Duarte and Tomás 2014; Mühleip et al. 2017; Yadav et al. 2017; Miranda-Astudillo et al. 2018b; Montgomery et al. 2018). In the present work, we studied the consequences of these atypical structures on the supramolecular association of the OXPHOS complexes in *E. gracilis* by native electrophoresis and single-particle electron microscopy, additionally, the in-vitro oxygen consumption activity of the purified respirasome complex (i.e. I/III_2_/IV) was determined.

## Materials and methods

### Algal strain, growth conditions and mitochondria isolation

*E. gracilis* (SAG 1224–5/25) was obtained from the University of Göttingen (Sammlung von Algenkulturen, Germany). Cells were grown in liquid mineral Tris-minimum-phosphate medium (TMP) pH 7.0 supplemented with a mix of vitamins (biotin 10^−7^%, B12 vitamin 10^−7^% and B1 vitamin 2 × 10^−5^% (*w*/*v*)). Ethanol 1% was used as carbon source. The cultures were grown in the dark under orbital agitation at 25 °C and collected in the middle of the logarithmic phase. Mitochondria were prepared as described in (Yadav et al. 2017) and stored at −80 °C until use. Protein concentration was determined by the Bradford method (Biorad).

### Native and denaturing protein electrophoresis

All steps were performed at 4 °C. n-dodecyl-β-D-maltoside (DDM, Sigma), digitonin (Sigma) and the synthetic drop-in substitute for digitonin glyco-diosgenin (GDN101, Anatrace) were used for the solubilization in a 4.0, 8.0 and 8.0 g detergent/mitochondrial protein ratio, respectively. Final concentrations of detergent were 3.2% or 6.4% in solubilization buffer (SB) containing 50 mM Tris-HCl, 1.5 mM MgSO4, 100 mM NaCl, 10% glycerol, 1 mM phenylmethylsulfonyl fluoride (PMSF) and 50 μg/mL tosyl-lysyl-chloromethylketone (TLCK) (pH 8.4). The mixture was incubated with gentle stirring for 30 min, and centrifuged at 90,000×g for 30 min as in (Miranda-Astudillo et al. 2018b). After discarding the insoluble material, the solubilized complexes were subjected to BN-PAGE in 3%–10% acrylamide gradient gels (Schägger 1994a), 0.05% digitonin was added in the acrylamide gradient if digitonin-extracted sample was loaded (Wittig and Schägger 2005).

To determine the molecular masses of the protein bands, the well characterized mitochondrial complexes from the chlorophycean alga *Polytomella* sp. were used as molecular mass markers (Miranda-Astudillo et al. 2018a). The logarithm of the distance migrated from each complex was interpolated into a Log (distance migrated) versus size (kDa) regression of the molecular markers (R^2^ = 0.9939) (Fig. S1 Suppl. Information). *In-gel* ATPase and complex I activities were carried out as in (Yadav et al. 2017; Miranda-Astudillo et al. 2018a). Denaturing 2D SDS Tricine-PAGE was carried out in 12% polyacrylamide gels as reported (Schägger 1994b). Two dimensional BN/BN-PAGE gels were carried out as previously described (Schägger and Pfeiffer 2001; Wittig and Schägger 2008).

### Respirasome complex (I/III_2_/IV) purification by liquid chromatography

All steps were performed at 4 °C. Thirty milligrams of mitochondria were solubilized with GDN101 as described above. The mixture was incubated with gentle stirring for 60 min, and centrifuged at 90,000×*g* for 30 min. The supernatant was diluted in 3 volumes of SB without NaCl and supplemented with GDN101 0.01%. The sample was loaded onto an anion exchange column (Source 15Q 5/50, Volume column (VC): 1 mL) connected to an ÄKTA monitor UPC-900 Workstation (GE Healthcare Life Sciences) equilibrated with the same buffer and washed until a constant baseline was obtained. The bound proteins were eluted with a linear 0–500 mM NaCl (20 VC) in the same buffer supplemented with 0.01% GDN, 0.5 mL fractions were collected and analyzed by BN-PAGE.

The fractions corresponding to the respirasome (I/III_2_/IV) together with the respiratory supercomplexes (III_2_/IV_1–2_) were pooled and concentrated with an Amicon Ultra-15 Centrifugal Filter 100 kDa (EMD Millipore) to a final volume of 500 μL and injected to a Superose 6 10/300 (GE Healthcare Life Sciences) previously equilibrated with SB buffer containing NaCl 200 mM and GDN 0.01%. The elution was carried out at 0.25 mL/min, 0.5 mL fractions were collected and visualized by BN-PAGE. The samples enriched with mitochondrial respirasome complex were pooled and stored at −70 °C until use.

### Differential spectroscopy of the purified respirasome

Absorption spectra from purified respirasome were measured at 25 °C in a Cary 60 UV-Vis spectrophotometer (Agilent Technologies). Differential spectrum was obtained as the sodium dithionite reduced spectrum minus the potassium ferricyanide oxidized spectrum as described in (Mukai et al. 1989).

### Oxygen consumption of the purified respirasome

Oxygen consumption was assessed in a YSI model 5300 oxygraph equipped with a Clark-Type electrode as described in (Miranda-Astudillo et al. 2018a). The reaction vessel was a 200 μL water-jacketed chamber maintained at 30 °C. The activity buffer contained MOPS 50 mM, NaCl 100 mM, GDN 0.01% (pH 7.2). NADH 5 mM was used as electron donor and 2,3-dimethoxy-5-methyl-p-benzoquinone (5 mM) was used to complete the electron transfer chain. The reaction was initiated with the addition of 100 μg of the purified respirasome. Specific inhibitors for complex I (rotenone 500 μM) and complex III (antimycin A 100 μM and myxothiazol 100 μM) were evaluated.

### Supercomplexes structure modelling

The crystal structures from chicken dimeric complex III (PDB: 4U3F (Hao et al. 2015)) and the monomeric bovine complex IV together with the cytochrome *c* (PDB: 5IY5 (Shimada et al. 2017)) were aligned with the corresponding chains in the mammalian respirasome model (PDB 5GUP (Wu et al. 2016) and fit into the density in the electronic map obtained from mammalian respirasome (EMD: 9539 (Wu et al. 2016)). Both structures were used together as a unique coupled model to interpret the projections from the *Euglena* III_2_/IV SC.

The cryo-EM structures from dimeric ATP synthase from *E. gracilis* (PDB: 6TDU (Mühleip et al. 2019)) were fit inside the 27.5 Å 3D map from ribbon of ATP synthases (three dimers) obtained by electron cryotomography and subtomogram averaging from intact inner mitochondrial membranes (EMD-3559 (Mühleip et al. 2017)). All the structure fitting and the images were generated using the UCSF Chimera (https://www.cgl.ucsf.edu/chimera/) (Pettersen et al. 2004).

## Results

### ATPase oligomers and respiratory supercomplexes in *E. gracilis*

**Fig. 1:**
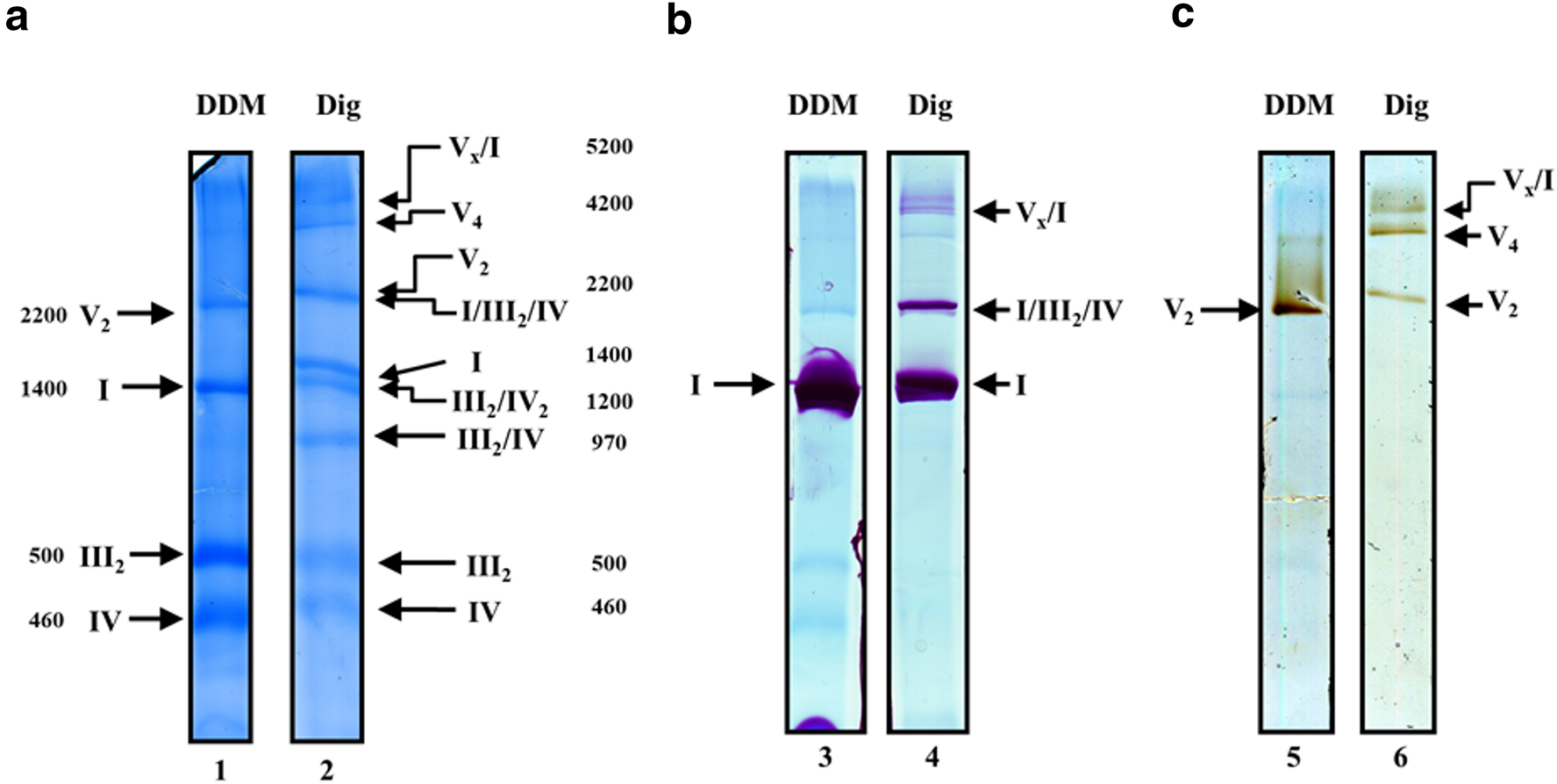
ATPase oligomers and respiratory supercomplexes in *E. gracilis*. Isolated mitochondria were solubilized with the indicated detergent: n-dodecyl-β-D-maltoside (DDM) at 4.0 g/ g protein or digitonin (Dig) at 8.0 g/g protein, after removing the insoluble material, each sample was resolved by BN-PAGE in a 3–10% polyacrylamide gradient gel. **a** Coomassie-stained gel showing the main bands with DDM- and digitonin-solubilization. **b** *In-gel* NADH-dehydrogenase activity; the BN-gel was incubated in the presence of NADH and Nitro blue tetrazolium chloride (NBT). **c** Detection of *in-gel* ATPase activity. The gel was incubated with ATP, MgSO_4_ and Pb(NO_3_)_2_. The determined molecular mass (kDa) of each isolated complex or supercomplexes is indicated. Nomenclature used: I, III_2_ and IV for the corresponding mitochondrial complexes, V_2_ and V_4_ for the dimeric and tetrameric ATP synthase respectively. Supercomplexes were: III_2_/IV, III_2_/IV_2_, the so-called “respirasome” association I/III_2_/IV and the putative V_x_/I association, their stoichiometries are indicated as subindexes

Mitochondria from dark-grown *E. gracilis* were treated with mild detergents: 4.0 g n-dodecyl-β-D-maltoside (DDM)/g protein (3.2% *w*/*v*) or 8.0 g digitonin/g protein (6.4% w/v). The native protein complexes were then subjected to a 3–10% acrylamide gradient BN-PAGE. When DDM was used to solubilize the complexes, four prominent bands ranging from 460 to 2200 kDa were found in the Coomassie-stained gel (Fig. 1 lane 1). They correspond to the dimeric complex V (V_2_), monomeric complex I (I), dimeric complex III (III_2_), and monomeric complex IV (IV) (Perez et al. 2014; Yadav et al. 2017; Miranda-Astudillo et al. 2018b). By contrast, when digitonin was used, four additional main bands ranging between 970 kDa and 5.2 MDa were observed (Fig. 1 lane 2), which may represent supramolecular associations between the OXPHOS complexes (i.e. supercomplexes, SC). Additionally, we tested a synthetic digitonin substitute (GDN101, Anatrace). Compared to digitonin, this substitute had a different impact on SCs solubilization: the 0.97, 1.2, 4.2 and 5.2 MDa bands are fainter while the 2.2 MDa band is more prominent (Fig. S2 lane 2 Suppl. Information).

### Composition of *E. gracilis* supercomplexes

To get insight into the composition of the newly identified protein bands, lanes with digitonin- and DDM- solubilized complexes were used to perform *in-gel* staining for complex I and complex V activities (Fig. 1b and c). In the case of digitonin solubilization, three complex I-stained bands were observed (1.4, 2.2 and 5.2 MDa, respectively), the lower and more intense band matches with the monomeric complex I in DDM lane (1.4 MDa) while the band above (2.2 MDa) probably corresponds to the association of complex I with dimeric complex III or complex IV. This band very often co-localized with dimeric complex V (Fig. 1 lanes 4 and 6). On the other hand, three digitonin-solubilized bands exhibited ATPase activity. The lower one has the same molecular mass as the V_2_ in DDM lane (2.2 MDa), the second prominent band might correspond to a tetrameric complex V (4.2 MDa) which is also observed as a faint band in DDM solubilisation (Fig. 1c lanes 5 and 6). Notably, the faint upper band at 5.2 MDa presents both complex V and complex I activity staining. Finally, two digitonin-solubilized bands without CI or CV activity are observed below complex I and above dimeric complex III at 1200 kDa and 970 kDa (Fig. 1 lanes 1 and 2), and may correspond to the previously described associations of complex III with IV (III_2_/IV and III_2_/IV_2_) in *Euglena* mitochondria (Perez et al. 2014). To discard that these supramolecular associations involving complex I or complex V are due to an incomplete solubilisation, *Euglena* mitochondria were solubilized with increasing digitonin concentrations (up to 12 g digitonin/g protein, 9.6% *w*/*v*). All the associations involving CI or CV described above, including putative SC I + III + IV and 5.2 MDa bands were stable at the highest concentration of detergent (Fig. S3, *red arrow,* Suppl. Information).

**Fig. 2:**
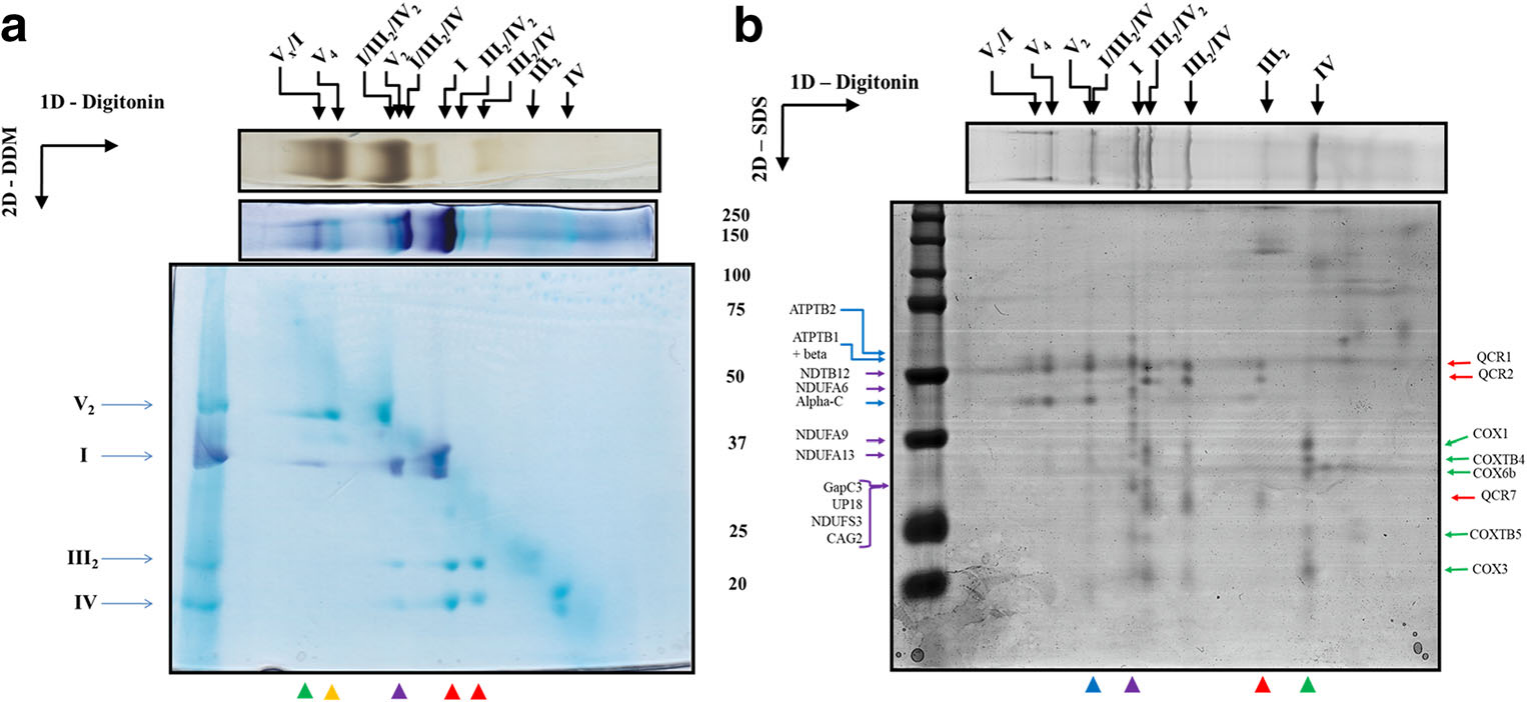
Two-dimensional resolution of OXPHOS complexes and supercomplexes in *E. gracilis* mitochondria. **a** U*pper panels:* The OXPHOS complexes and supercomplexes from *Euglena* mitochondria were solubilized using digitonin (Dig) and separated by BN-PAGE followed by *in-gel* NADH-dehydrogenase and ATPase activities. *Lower panel:* NADH-dehydrogenase activity stain of two-dimensional gel from digitonin-extracted complexes and supercomplexes, the isolated spots show the complexes present in each supercomplex. III_2_/IV and III_2_/IV_2_ (*red arrowheads*), the respirasome (I/III_2_/IV, *purple arrowhead*), V_4_ oligomer (*yellow arrowhead*) and the V_x_/I association (*green arrowhead*), isolated complexes were used as molecular mass markers (*left lane*). **b** The OXPHOS complexes and supercomplexes from *Euglena* mitochondria were solubilized using digitonin (*upper lane*) and separated by BN-PAGE. *Lower panel:* Two-dimensional SDS-tricine gel from digitonin-extracted complexes and supercomplexes. Representative subunits of each complex NDUFS3/NDUFA6/NDUFA9/NDUFA13/NDTB12/GapC3 for CI (*purple arrows*), QCR1/QCR2/QCR7 for CIII (*red arrows*), COX1/COX3/COXTB4/COXTB5/COX6b for CIV (*green arrows*) and ATPTB1/ATPTB2/Alpha-C/Beta for CV (*blue arrows*) are indicated. Molecular masses from the molecular mass marker are indicated on left side

To further characterize the composition of these stable supramolecular associations, a second BN-PAGE with 0.02% of DDM in the cathode buffer was performed on the acrylamide lanes comprising separated digitonin- or DDM- solubilised complexes. All DDM-solubilized complexes separated as individual complexes in the 1D BN-PAGE are now found on a diagonal according to their molecular masses in the 2D gel (Fig. S4, Suppl. Information). In contrast, the digitonin-solubilized SCs are dissociated by the DDM present in the cathode buffer during the 2D BN-PAGE (Fig. 2a). As expected, individual complexes dissociated from SCs migrated below the diagonal at positions in the gel that correspond to the migration distances of the four native DDM-solubilized respiratory complexes I, III_2_, IV or V_2_ (shown on the left part of Fig. 2a). In the case of complex I, its identity was further confirmed by *in-gel* staining activity. The band between the monomeric complex I and dimeric complex V bands (Fig. 2a, *purple arrowhead*) comprises CI, CIII, and CIV and might thus correspond to the so-called “respirasome” with an I/III_2_/IV stoichiometry (2.2 MDa). The 4.2 MDa spot only comprises dimeric complex V and may thus corresponds to a tetrameric complex (V_4_) (Fig. 2a*yellow arrowhead*). In some cases, two faintly ATPase activity bands are also observed in the 1D gel below the V_2_ band (Figs. 2a and S4, *upper lanes*), these bands (marked as *a* and *b*) correspond to partial dissociation of the dimeric complex V that is also observed when the purified V_2_ complex is incubated in presence of DDM (Fig. S5, Suppl. Information). Finally, a co-migration involving CI and CV_2_ is visible, interestingly, CI and CV activities are present in this 5.2 MDa band but no evidence of CIII and CIV presence after the Coomassie blue staining was observed (data not shown), this opens the possibility that these two complexes form a larger supercomplex (Fig. 2a, *green arrowhead*).

To further confirm the composition of these SCs, the digitonin-extracted complexes were subjected to a 2D BN/SDS-PAGE. Based on the electrophoretic profile of subunits constitutive for each isolated complex (Fig. S6, Suppl. Information) (Perez et al. 2014; Yadav et al. 2017; Miranda-Astudillo et al. 2018b), several subunits representative of the individual complexes were identified in each of the SCs (e.g. NDTB12, NDUFA6, NDUFA9, NDUFA13 and GapC3 of CI, QCR1, QCR2 and QCR7 of CIII, COX1, COX3, COXTB4, COX6b and COXTB5 of CIV and ATPTB1, ATPTB2 Alpha-C and Beta of CV) (Fig. 2b). In the largest 5.2 MDa band, CI components are barely visible. This suggests that this band is dominated by a CV oligomer (V_5_ or V_6_). Relatively to complex III components, complex IV subunits are more abundant in the 1200 kDa band than in the 970 kDa band. This observation is in line with a greater complex III: complex IV stoichiometry in the 1200 kDa band of the two dimensional BN-PAGE/BN(+DDM)-PAGE (Fig. 2a, *red arrowheads*), suggesting the existence of two CIII/CIV SCs with two different stoichiometries: III_2_/IV or III_2_/IV_2_ for the 970 and 1200 kDa bands, respectively. Taken together, our results show that four isolated complexes and five larger SCs can be isolated from *Euglena* mitochondria. The proposed stoichiometry for each SC is also supported by the good correspondence between the determined and the expected molecular masses for all the SCs (linear regression coefficient R^2^ = 0.9971) (Fig. S7).

### Purification of the Euglenoid respirasome

**Fig. 3:**
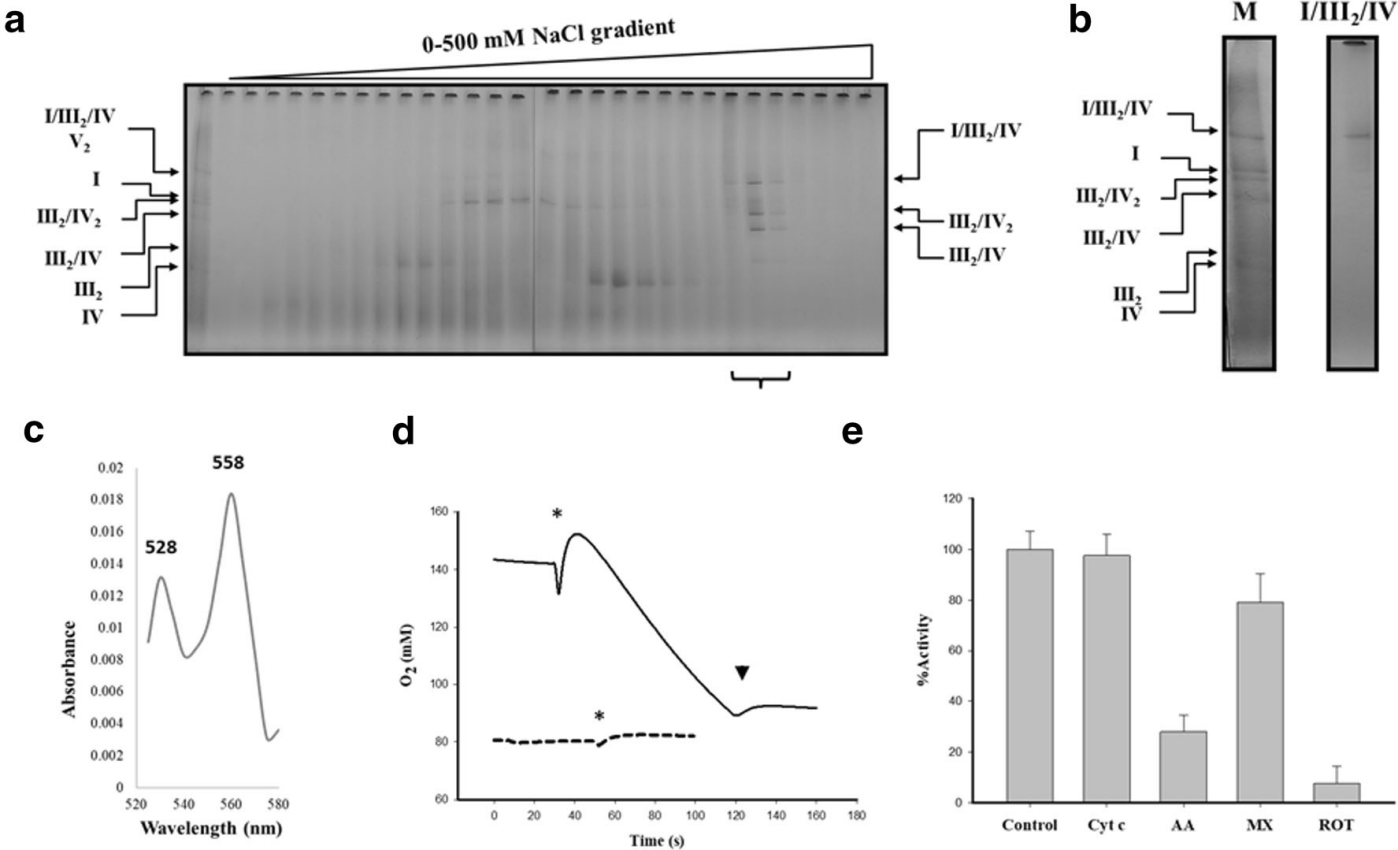
Purification of the Euglenoid respirasome (I/III_2_/IV) by ion exchange/size exclusion chromatography and in-vitro oxygen consumption. Thirty milligrams of mitochondria were solubilized with glyco-diosgenin (GDN101) and loaded into an anion exchange column, then eluted with a 0–500 mM NaCl linear gradient. **a** BN- PAGE from the eluted 0.5 mL fractions, fractions containing III_2_/IV, III_2_/IV_2_ and I/III_2_/IV SCs (*lower bracket*) were concentrated and subjected to size exclusion column. **b** BN-PAGE from the pure respirasome (I/III_2_/IV) and the sample load onto the column (M), the identities of the SCs are indicated. **c** Differential redox absorption spectrum (520–580 nm) of purified respirasome was obtained as the sodium dithionite reduced spectrum minus the potassium ferricyanide oxidized spectrum. Alpha (558 nm) and beta (528 nm) peaks are signaled. **d** Oxygen consumption of the purified respirasome. The purified respirasome was incubated in the presence of NADH as electron donor (segmented line). External oxidized 2,3-Dimethoxy-5-methyl-p-benzoquinone was added (continuous line). The asterisk indicates the addition of the protein sample and the arrowhead indicates the addition of the complex I inhibitor rotenone. The lines were moved along the y axis for clarity. **e** Effect of external cytochrome *c* and inhibitory effect of antimycin A, myxothiazol and rotenone over the purified *Euglena* respirasome. The values represent the mean of three independent experiments and the bars represent the standard deviation

To further characterize the euglenoid respirasome, the GDN101-extracted I/III_2_/IV SC was purified by a two-step chromatographic procedure (see Material and methods section 2.3 for further details). The fractions containing associations between CI, CIII and CIV were enriched after the anion exchange chromatography (Fig. 3a, *lower bracket*), and a purified fraction containing the three complexes I/III_2_/IV association was obtained in the size exclusion chromatographic step (Fig. 3b). The redox differential absorption spectrum of the purified respirasome shows 528 nm and 558 nm peaks (Fig. 3c) which are typical of *Euglena* cytochrome *c* (*c*-558), as reported previously (Pettigrew et al. 1975; Mukai et al. 1989). To estimate the activity of this purified respirasome, in-vitro oxygen consumption was assayed upon addition of NADH as an electron donor. No oxygen consumption occurred at this point (Fig. 3d*segmented line*) indicating either the interruption of the electron transport (e.g. loss of electron carriers by the detergent effect) or the damage/loss of function of the complexes during the purification procedure. Nevertheless, addition of external 2,3-dimethoxy-5-methyl-p-benzoquinone together with NADH led to a substantial oxygen consumption (Fig. 3d*continuous line*). Further addition of external horse cytochrome *c* did not enhance this in-vitro activity. This activity was also inhibited by rotenone (CI inhibitor) and antimycin A (CIII inhibitor) (Fig. 3e). In contrast, it was barely affected by the presence of myxothiazol (CIII inhibitor) (Fig. 3e).

## Discussion

### Canonical association between complexes III & IV in *E. gracilis*

**Fig. 4:**
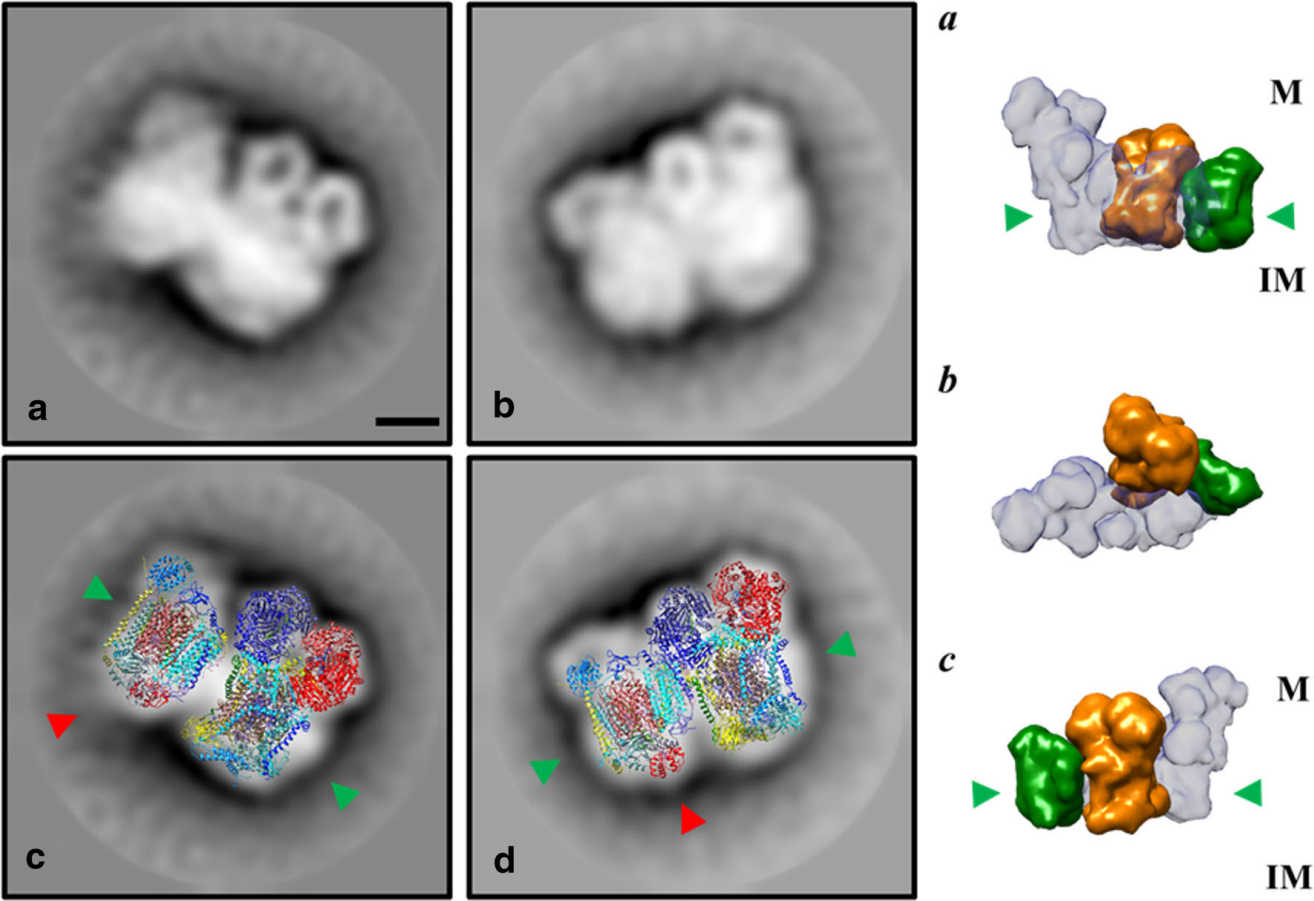
2D Projection maps of III_2_/IV supercomplex from *E. gracilis* obtained by single particle averaging. *Left panels*: A fraction containing both complexes was obtained after a two-step chromatographic procedure in presence of β-dodecyl-n-maltoside and analyzed by TEM (**a** and **b**). Overlap of the coupled model (see material and methods point 2.6 for further details) built with chicken dimeric complex III (pdb: 4U3F (Hao et al. 2015)) and the monomeric bovine complex together with the cytochrome *c* (pdb: 5IY5 (Shimada et al. 2017)) over the TEM images was performed (**c** and **d**). The membrane region is indicated by the green arrowheads, the cytochrome *c* binding site is indicated with red arrowheads. *Right panels* (a-c): model showing the position of the III_2_/IV supercomplex inside the mammalian respirasome map (EMD: 9539 (Wu et al. 2016)). Orange: dimeric complex III, green monomeric complex IV, light grey: monomeric complex one. The membrane region is signaled (*green arrowheads*), topology of this supercomplex in the mitochondrial inner membrane is signaled (M: matrix, IM: intermembrane space). The scale bar is 10 nm

It has been recently shown that the OXPHOS complexes I, III_2_, IV and V_2_ from *E. gracilis* present atypical subunits which lead to characteristic structural features such as extra domains in complexes I, IV and V_2_, when comparing them with their homologs in classical model organisms (e.g. mammals and yeast) (Yadav et al. 2017; Miranda-Astudillo et al. 2018b). Digitonin is a widely-used detergent to study the supramolecular association in mitochondrial complexes from several species (Paumard et al. 2002; Bultema et al. 2009; Dudkina et al. 2011). This detergent is known to favour the native associations between membrane complexes from different organelle sources (Schägger 2002; Vonck and Schäfer 2009; Benson et al. 2015). When *Euglena* mitochondria were treated with digitonin or its synthetic substitute GDN101, four main additional supramolecular associations are observed (Fig. 1). Among them, three correspond to supercomplexes involving complexes III and IV. The association of *Euglena* complexes III and IV was already observed in native gels with low DDM concentration or digitonin extractions (Perez et al. 2014). Based on their estimated molecular mass of 2200, 1200 and 970 kDa, and on the relative abundance of each complex, these SCs probably correspond to I/III_2_/IV, III_2_/IV_2_ and III_2_/IV, respectively. Structures of both I/III_2_/IV, and III_2_/IV_2_ SCs have been already characterized in mammals, yeast and plants (Davies et al. 2018; Rathore et al. 2019). We reanalysed transmission electron microscopy (TEM) images obtained from an enriched fraction containing both complexes III and IV purified in presence of DDM (Miranda-Astudillo et al. 2018b). Further single particle analysis from this fraction revealed two additional classes of images that may correspond to an association between complexes III and IV (Fig. 4, *upper panels*). The atypical “helmet-like” domain of the *Euglena* cytochrome *c* oxidase (Miranda-Astudillo et al. 2018b) is however not visible in the two projections. This atypical extra domain exposed to the intermembrane side (p side) (Fig. 4, *red arrowheads*) was proposed to build a specific cavity for the endogenous cytochrome *c* (Miranda-Astudillo et al. 2018b). The III_2_/IV model built from the electronic density map recently obtained from mammalian respirasome (EMD: 9539 (Wu et al. 2016)) (Fig. 4, *right panels*) explains both EM projections (Fig. 4, *lower panels*). This suggests that the overall structural association between complex III and IV described for porcine (Wu et al. 2016), bovine (Davies et al. 2018) and yeast (Heinemeyer et al. 2007) mitochondria is conserved in *E. gracilis*, extending the previous proposed idea that the fundamental features of the supramolecular organisation (i.e., structure, composition, stoichiometry) of the respiratory complexes were conserved in lineages beyond classical mammals, fungi, and flowering plants models (Krause et al. 2004), supporting the idea of a common ancestry of these organelles (Margulis 1971).

### The purified respirasome lost the dedicated quinone pool but conserved the endogenous euglenoid cytochrome c

After both steps of anion exchange chromatography and size exclusion chromatography, a stable I/III_2_/IV respirasome complex was separated from the smaller SCs (III_2_/IV_1_ and III_2_/IV_2_). This is, to our knowledge, the first purification of a complete and functional respirasome from an organism beyond the classical model organisms (i.e., mammals, yeasts, green plants). The presence of the endogenous cytochrome *c* bound to the purified respirasome was evidenced (Fig. 3c). As a result, this respirasome was capable of transferring electrons from NADH to oxygen provided that only external quinones were added. This contrasts with the in-vitro reconstituted respirasome from the chlorophycean alga *Polytomella* sp. which requires further addition of both external electron carriers (quinone and cytochrome *c*) (Miranda-Astudillo et al. 2018a). Recently, it was also shown that oxygen consumption in presence of NADH of the purified respirasome from *U. maydis* is enhanced when external electron carries were added (Reyes-Galindo et al. 2019). Our observation thus suggests that association between endogenous cytochrome *c* and the respirasome in *E. gracilis* is more stable than in other species. Electron density corresponding to cytochrome *c* is observed in the III_2_/IV_1_ SC projections (Fig. 4*red arrow heads*). The presence of an identified helmet-like extra domain in isolated *Euglena* complex IV (Miranda-Astudillo et al. 2018b) might prevent the release of cytochrome *c* from the respirasome. Incidentally, the existence of such an unusual binding cavity for cytochrome *c* would be in line with previous works that showed that *Euglena* CIV is unable to used cytochrome *c* from other species (Collins et al. 1975; Brönstrup and Hachtel 1989).

Another major difference is the insensitivity of *E. gracilis* complex III to myxothiazol (Moreno-Sánchez et al. 2000; Perez et al. 2014). Our results (Fig. 3e) are in line with the inhibitors sensitivity previously described for fresh mitochondria (Mukai et al. 1989). It has been suggested earlier that the putative presence of a previously proposed additional CIII-like complex (Moreno-Sánchez et al. 2000) might explain this differential sensitivity. In this respect, we proposed that the presence of atypical QCR1/2 orthologs in *E. gracilis* (QCRTB1/2) along with the lack of the conserved QCR8 subunit, and the presence of an atypical QCR7 N-terminal extension (~ 100 residues) may affect the binding site of myxothiazol (Perez et al. 2014; Miranda-Astudillo et al. 2018b). Recently, Krnáčová et al. (2015), showed a myxothiazol- and antimycin- sensitive respiration (~50%) in *E. gracilis* but only one type of CIII has been identified in their study (Krnáčová et al. 2015) as in ours (Perez et al. 2014; Miranda-Astudillo et al. 2018b). These differences of sensitivity to myxothiazol and antimycin A can perhaps be explained by a difference in accessibility of the inhibitors to their binding site depending on the oligomeric state (monomer, dimer, supercomplex), which can be different depending on the culture conditions, the type of samples and the preparative methods (cells, mitochondria, isolated complexes), or their storage conditions (frozen, fresh).

The substrate channeling, specially the dedicated quinol pool, is one of the major advantages proposed for the respirasome formation. Nevertheless, recent spectroscopic and kinetic experiments performed in mammalian mitochondria refute this idea and points towards the existence of an universally accessible ubiquinone/ubiquinol pool that is not partitioned or channelled (Blaza et al. 2014; Fedor and Hirst 2018). These results together with the absence of known supercomplex-mediating factors in extensive structural data of isolated complexes (Davies et al. 2011, 2018; Dudkina et al. 2011) together with the ability to reconstitute functional SCs from isolated complexes (Bazán et al. 2013; Miranda-Astudillo et al. 2018a) stand up for a structural role of these SCs specially working in favour of CI stability (Acin-Perez and Enriquez 2014). Additionally, specific interactions between the complexes may protect against non-specific aggregation in the high protein concentration of the mitochondrial inner membrane (Blaza et al. 2014) and promote higher diffusion rates of the membrane embedded quinones (Kirchhoff 2014; Fedor and Hirst 2018). Putative respiratory strings, formed by an association of respirasomes have been observed by the study of the freeze-fractured and deep-etched inner mitochondrial membranes from *Paramecium multimicronucleatum* (Allen et al. 1989), and also have been proposed to be present in mammalian and plant mitochondria based on electron microscopy and native electrophoresis analysis (Wittig et al. 2006b; Bultema et al. 2009; Nubel et al. 2009; Wittig and Schägger 2009; Dudkina et al. 2010) where I_2_/III_2_/IV_2_ and I_2_/(III_2_)_2_/IV_2_ SCs should work as building blocks according to circular or linear models respectively (Bultema et al. 2009; Letts et al. 2016; Guo et al. 2018). Our results when digitonin or GDN101 are used for the solubilisation of membranes indicated that a large amount of CI, CIII and CIV are involved in SCs. This suggests the existence of respirasome strings as well in *Euglena*.

### Tetrameric stable ATP synthase from *E. gracilis*

The dimeric mitochondrial ATP synthases of opisthokonts (i.e. mammals and fungal enzymes) easily dissociates into monomers in presence of DDM when subjected to BN-PAGE (van Lis et al. 2003, 2007; Wittig et al. 2006a). In contrast, *Euglena* DDM-extracted ATP synthase remains as a stable dimer and even a remnant of the tetrameric form (V_4_) is detectable (Fig. 1c, lane 5). Similarly, a highly stable DDM-solubilized tetrameric ATP synthase has already been observed in mitochondria of chlorophycean algae (Miranda-Astudillo et al. 2018a). In contrast, no tetrameric complex from opisthokonts (*i.e* metazoan and or fungi) has been observed in presence of DDM. Oligomeric forms have been however observed when digitonin is used to extract the complex (*e.g* in bovine (Strauss et al. 2008; Wittig and Schägger 2009), porcine (Gu et al. 2019) and yeast (Thomas et al. 2008; Habersetzer et al. 2013)). The cryo-EM structure of the mammalian tetrameric enzyme was recently obtained (Gu et al. 2019). In an H-shape, two dimers are bound mainly by their membrane sector as previously proposed based on crosslinking experiments and 2D microscopy images from the isolated tetramers (Thomas et al. 2008; Habersetzer et al. 2013). Additionally, two dimers of the inhibitory subunit IF_1_ link the F_1_ sectors from the adjacent dimers (Gu et al. 2019). This ATPase oligomerization leads to ATPase ribbons formation (Strauss et al. 2008; Blum et al. 2019).

**Fig. 5:**
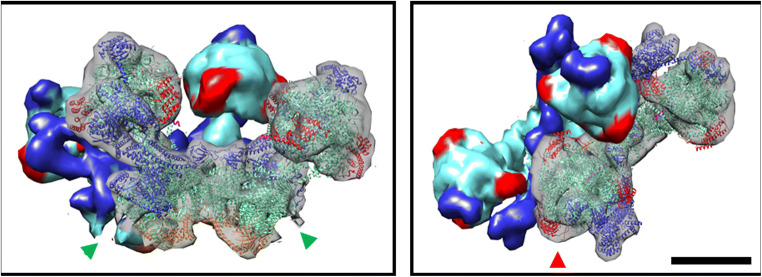
Putative tetrameric structure of the *E. gracilis* ATP synthase. Putative tetrameric structure of the *E. gracilis* ATP synthase based in the electron cryotomography images from intact mitochondria membranes (Mühleip et al. 2017). The externally located peripheral stalks are shown in purple, the inter membrane space density below the *c*-ring is shown in orange, the F_1_/central rotor sector is shown in cyan, the membrane region is signalled (*green arrowheads*) and the position of the euglonoid specific subunit *p18* is indicated (*red arrowheads*), the structure of the *Euglena* ATP synthase dimer (PDB: 6TDU (Mühleip et al. 2019)) is fitted inside the electron density for one dimer. The scale bar is 10 nm

To get some insight into the structure of the tetrameric form of *Euglena* ATP synthase, the recently obtained cryo-EM structure of the *Euglena* ATP synthase dimer (Mühleip et al. 2019) were fitted inside the 27.5 Å 3D map (EMD: 3559) from *Euglena* ATP synthase ribbon determined by electron cryotomography and subtomogram averaging from intact mitochondrial membranes (Mühleip et al. 2017). This comparison let us propose a putative structure of the *Euglena* tetrameric complex V where the dimer-dimer interaction is present mainly at the membrane level (Fig. 5, *green arrowheads*), and where the external peripheral stalks together with one of the *p18* subunits face outside of the ribbon (Fig. 5, *red arrowheads*). The possible role of the euglenoid subunit *p18* (Zíková et al. 2009; Perez et al. 2014; Yadav et al. 2017) remains obscure, nevertheless, its structural role could be related to its exposed side in the ATP synthase ribbon. The possible dimer-dimer contact zone should be also located mainly among the large membrane-spanning region described for *Euglena* enzyme (Mühleip et al. 2017; Yadav et al. 2017), especially because in the *Euglena* enzyme the IF_1_ peptide is binding the adjacent monomers inside one dimer (Mühleip et al. 2019) and not between adjacent dimers as described for the mammalian tetramer (Gu et al. 2019).

The angle formed between the CV membrane sector (F_O_) of the two monomers in *Euglena* dimer is much lower (40°) than in the dimers of opisthokonts (70°), so the effect of these dimers on cristae shape might be different. In this respect, lamellar cristae are found in the supergroup of opisthokonts, while tubular-shaped cristae typify the unicellular supergroup SAR, containing stramenopiles, alveolates, and rhizarians (Mühleip et al. 2016, 2021). Euglenids, members of the protistan supergroup Excavata, generally have discoidal cristae, which exhibit a paddle-like morphology (Kaurov et al. 2018). Electron cryotomography analyses on mitochondria from plants, mammals and yeasts showed that the dimeric ATP synthase rows locate along the cristae curvature, bending the membrane while the rest of the complexes are irregularly distributed confined to flat membrane regions (Davies et al. 2011, 2018). Some CI particles are however located beside the CV row in tomographic slices of isolated cristae membranes from bovine mitochondria (Davies et al. 2011). In *Euglena*, the comigration of a fraction of CI and oligomeric CV in BN-PAGE experiments, could reflect a similar partitioning in vivo. Similar comigration of CV together with respiratory complexes has been observed recently in pea mitochondria treated with digitonin (Ukolova et al. 2020).

### Conserved supercomplexes formation in *E. gracilis*

**Fig. 6:**
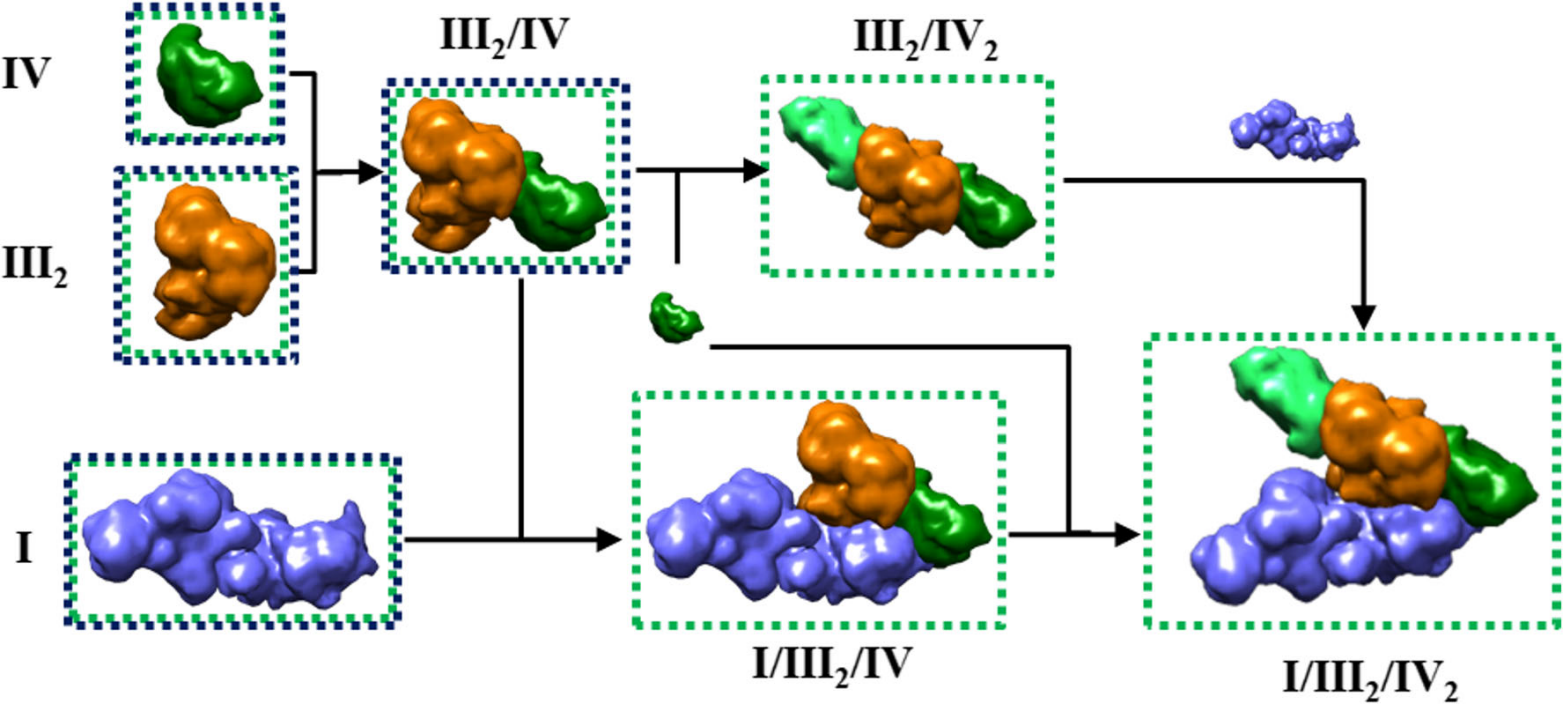
Assembly pathway of mitochondrial respirasome in *E. gracilis*. Schematic representation of the associations of complexes into supercomplexes. The species observed by BN-PAGE (I, III_2_, IV, V_2_, III_2_/IV, III_2_/IV_2_, I/III_2_/IV, I/III_2_/IV_2_) and TEM analysis (I, III_2_, IV, and III_2_/IV) from *Euglena* mitochondria ((Yadav et al. 2017; Miranda-Astudillo et al. 2018b), this work) are shown in green and dark blue boxes, respectively

The OXPHOS complexes from *E. gracilis* contain atypical subunits which lead to extra structural domains (Yadav et al. 2017; Miranda-Astudillo et al. 2018b). Nevertheless, they form classical III_2_/IV_1–2_ associations and the respirasome (I/III_2_/IV). The mammalian respirasome present four major structural pivots, two of them related with the CI/CIII_2_ association, one with CIII_2_/IV interface and the last with CI membrane extrinsic arm (Letts and Sazanov 2017), leaving a major structural role in the central position of CIII_2_ whose in situ arrangement is conserved between the opisthokonts and plant mitochondria (Davies et al. 2018). In this respect, despite that *Euglena* CIII presents four atypical subunits, the overall structure of the dimeric complex is conserved (Miranda-Astudillo et al. 2018b), and a canonical CIII_2_/IV arrangement is observed in DDM-extracted III_2_/IV supercomplex (Fig. 4). Additionally, *Euglena* III_2_/IV SC can bind a second monomeric CIV forming a III_2_/IV_2_ SC that may form a larger respirasome (I/III_2_/IV_2_) observed at 2450 kDa band (Fig. 2a, upper bands). This latter species is however less stable in our experimental conditions than the purified 2200 kDa respirasome (I/III_2_/IV) (Fig. 3b). Similarly, in situ observation inside the inner membrane of mammalian mitochondria showed that I/III_2_/IV SC is more abundant than I/III_2_/IV_2_ SC (Davies et al. 2018). Finally, the data obtained in the present work and in our previous works (Perez et al. 2014; Yadav et al. 2017; Miranda-Astudillo et al. 2018b) let us propose an assembly pathway for *Euglena* respirasome SC from OXPHOS complexes (Fig. 6) quite similar to the one described in other linages (Lobo-Jarne and Ugalde 2018), despite atypical subunit composition and additional structural domains of the oxidative phosphorylation complexes in *Euglena gracilis*.

## Supplementary Information

Figure S1 Estimated molecular masses for the *Euglena gracilis* supercomplexes. The logarithms of the molecular masses of previously characterized mitochondrial complexes from the colorless algae *Polytomella* sp. (◆) (V_6_, V_4_, V_2_, I, III_2_; see (Atteia et al. 2003; van Lis et al. 2003, 2005, 2007; Cardol et al. 2009; Miranda-Astudillo et al. 2018a) for details) were plotted against their migration distance in BN-PAGE. Then, the migration distances of the *Euglena* digitonin-extracted supercomplexes (○) were interpolated and their corresponding molecular masses inferred. The proposed stoichiometry and the molecular mass of each complex and supercomplex are indicated. (PNG 757 kb)

High Resolution Image (TIF 29236 kb)

Figure S2 ATPase oligomers and respiratory supercomplexes in *Euglena gracilis* extracted with non-ionic detergents. Isolated mitochondria were solubilized with digitonin (Dig) and its synthetic drop-in substitute glyco-diosgenin GDN101 (GDN) at 8.0 g/g protein. Final concentrations of detergent were 6.4%. After removing the insoluble material, each sample was resolved by BN-PAGE in a 3–10% polyacrylamide gradient gel. The determined molecular mass of each isolated complex or supercomplexes are indicated. Nomenclature used: I, III and IV for the corresponding mitochondrial complexes, V_2_ and V_4_ for the dimeric and tetrameric ATP synthase respectively. Supercomplexes were: III_2_/IV, III_2_/IV_2_, the so-called “Respirasome” association I/III_2_/IV and the putative V_x_/I association, their stoichiometries are indicated as subindexes. (PNG 1146 kb)

High Resolution Image (TIF 18828 kb)

Figure S3 Stability of the *Euglena gracilis* supercomplexes at increasing concentrations of digitonin. Isolated mitochondria were solubilized with increasing concentrations of digitonin. *Left panel*: *in-gel* NADH-dehydrogenase activity; the BN-gel was incubated in the presence of NADH and Nitro blue tetrazolium chloride (NBT). *Right panel*: BN-gel and detection of *in-gel* ATPase activity. The gel was incubated with ATP, MgSO_4_ and Pb(NO_3_)_2_. Numbers above represent the g of detergent/g of protein relation. (PNG 1880 kb)

High Resolution Image (TIF 30133 kb)

Figure S4 Two-dimensional resolution of OXPHOS complexes in *Euglena gracilis* mitochondria. *Upper lanes:* The OXPHOS complexes from *Euglena* mitochondria were solubilized using n-dodecyl-β-D-maltoside (DDM) and separated by BN-PAGE followed by *in-gel* NADH-dehydrogenase and ATPase activities. *Lower panel:* NADH-dehydrogenase activity stain two-dimensional gels from DDM-extracted complexes, the isolated spots in the diagonal show the complexes separated by their molecular masses in each dimension. Isolated complexes were used as molecular mass markers (*left lane*). (PNG 1702 kb)

High Resolution Image (TIF 29387 kb)

Figure S5 ATP synthase dissociation in *Euglena gracilis*. Purified dimeric ATP synthase was incubated in presence of n-dodecyl-β-D-maltoside (DDM) at room temperature for 10 min. Four species are observed which may correspond to dimeric enzyme V_2_, dimer without one F_1_ sector (band *a*), monomeric enzyme (band *b*), and the F_1_ sector. This assignment was done based on size. (PNG 1164 kb)

High Resolution Image (TIF 23977 kb)

Figure S6 Two-dimensional resolution of OXPHOS complexes in *E. gracilis* mitochondria. The OXPHOS complexes from *Euglena* mitochondria were solubilized using n-dodecyl-β-D-maltoside (DDM) (*upper lane*) and separated by BN-PAGE. *Lower panel:* Two-dimensional SDS-tricine gel from DDM-extracted complexes. Identified subunits by tandem mass spectroscopy of each complex are indicated (Yadav et al. 2017; Miranda-Astudillo et al. 2018b). Molecular masses from the molecular mass marker are indicated on left side. (PNG 2180 kb)

High Resolution Image (TIF 31356 kb)

Figure S7 Linear regression of calculated versus determined molecular masses in *Euglena gracilis* supercomplexes. (PNG 568 kb)

High Resolution Image (TIF 28797 kb)
