# Correction to: Supramolecular associations between atypical oxidative phosphorylation complexes of *Euglena gracilis*

**DOI:** 10.1007/s10863-021-09890-8

**Published:** 2021-05-07

**Authors:** H. V. Miranda-Astudillo, K. N. S. Yadav, E. J. Boekema, P. Cardol

**Affiliations:** 1 InBios/Phytosystems, Institut de Botanique, University of Liège, Liège, Belgium; 2 Present Address: Departamento de Biología Molecular y Biotecnología, Instituto de Investigaciones Biomédicas, Universidad Nacional Autónoma de México, Mexico City, Mexico; 3 Department of Electron Microscopy, Groningen Biological Sciences and Biotechnology Institute, University of Groningen, Groningen, the Netherlands; 4 Present Address: School of Biochemistry, University of Bristol, Bristol, BS8 1TD UK

**Correction to: Journal of Bioenergetics and Biomembranes.**

10.1007/s10863-021-09882-8

The original version of this article unfortunately a mistake. Table 1 should not be included in the proofs.

The original article has been corrected.

