# Supplementary figures and images for: Supramolecular associations between atypical oxidative phosphorylation complexes of Euglena gracilis
Source: J Bioenerg Biomembr. 2021 Mar 1;53(3):351–63. doi: 10.1007/s10863-021-09882-8 (PMC8124061; doi:10.1007/s10863-021-09882-8)

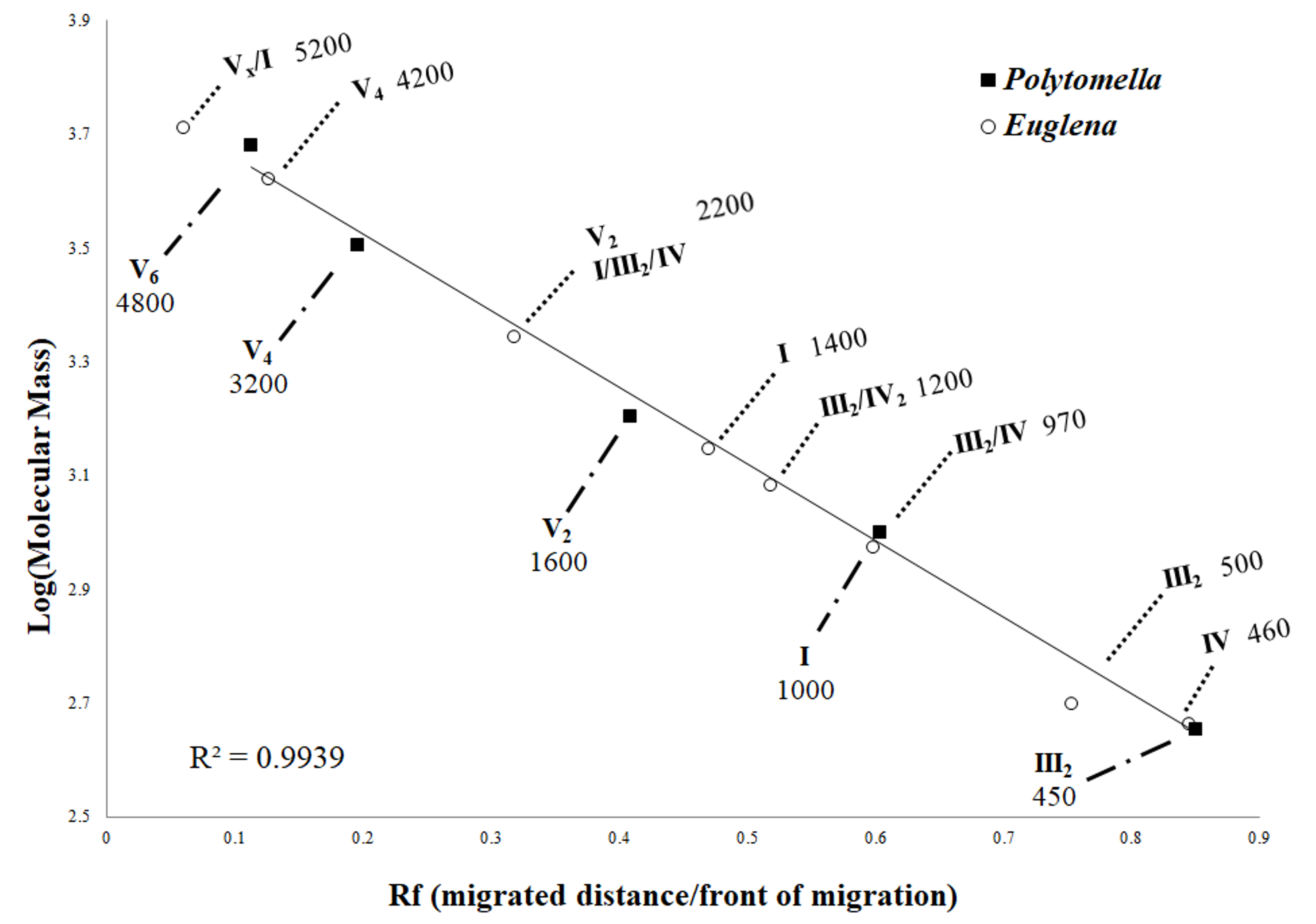

Supplement: Supplementary file 1 — Estimated molecular masses for the Euglena gracilis supercomplexes. The logarithms of the molecular masses of previously characterized mitochondrial complexes from the colorless algae Polytomella sp. (◆) (V6, V4, V2, I, III2; see (Atteia et al. 2003; van Lis et al. 2003, 2005, 2007; Cardol et al. 2009; Miranda-Astudillo et al. 2018a) for details) were plotted against their migration distance in BN-PAGE. Then, the migration distances of the Euglena digitonin-extracted supercomplexes (○) were interpolated and their corresponding molecular masses inferred. The proposed stoichiometry and the molecular mass of each complex and supercomplex are indicated. (PNG 757 kb) [file 10863_2021_9882_Fig7_ESM.png]

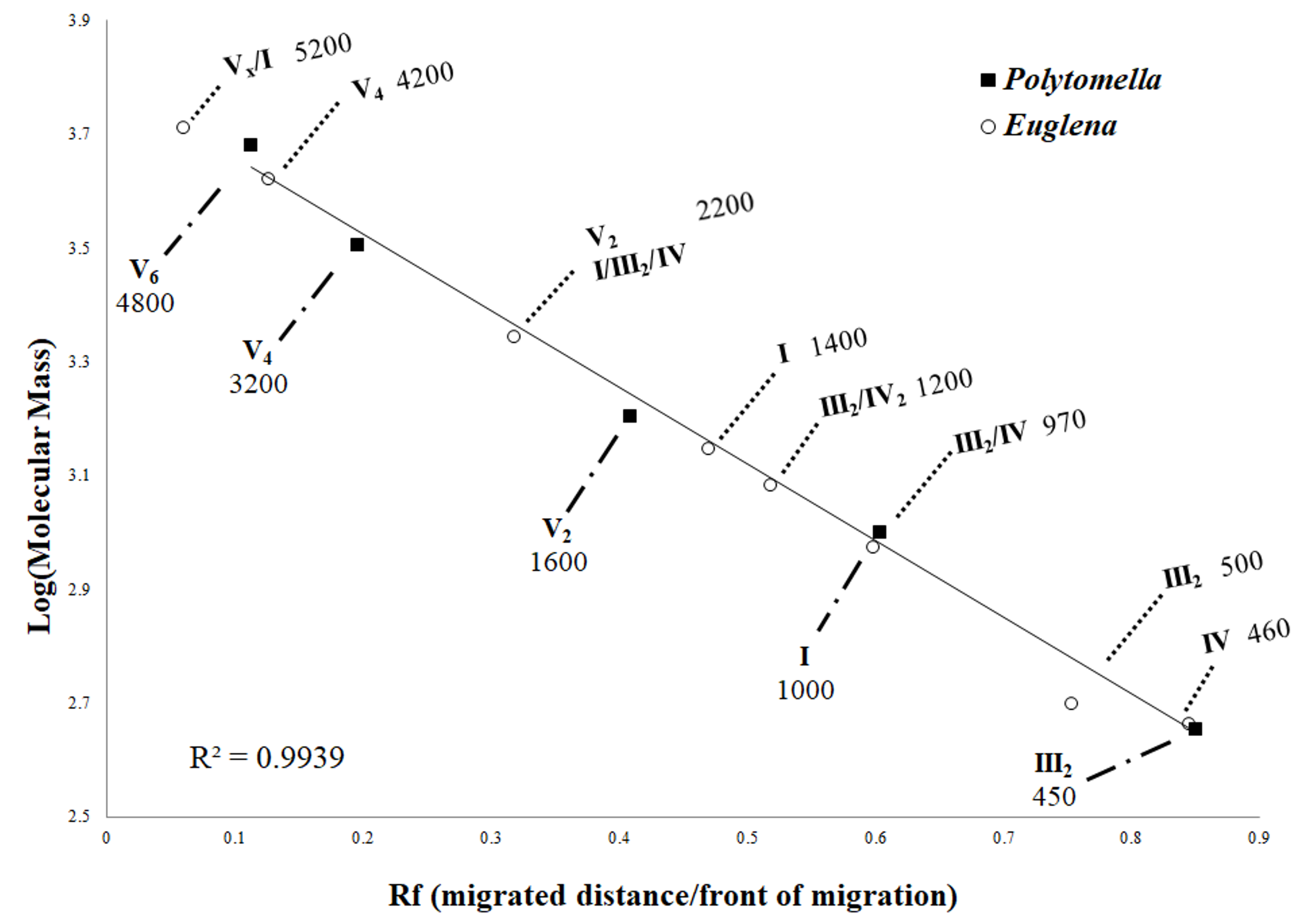

Supplement: Supplementary file 2 — High Resolution Image (TIF 29236 kb) [file 10863_2021_9882_MOESM1_ESM.tif]

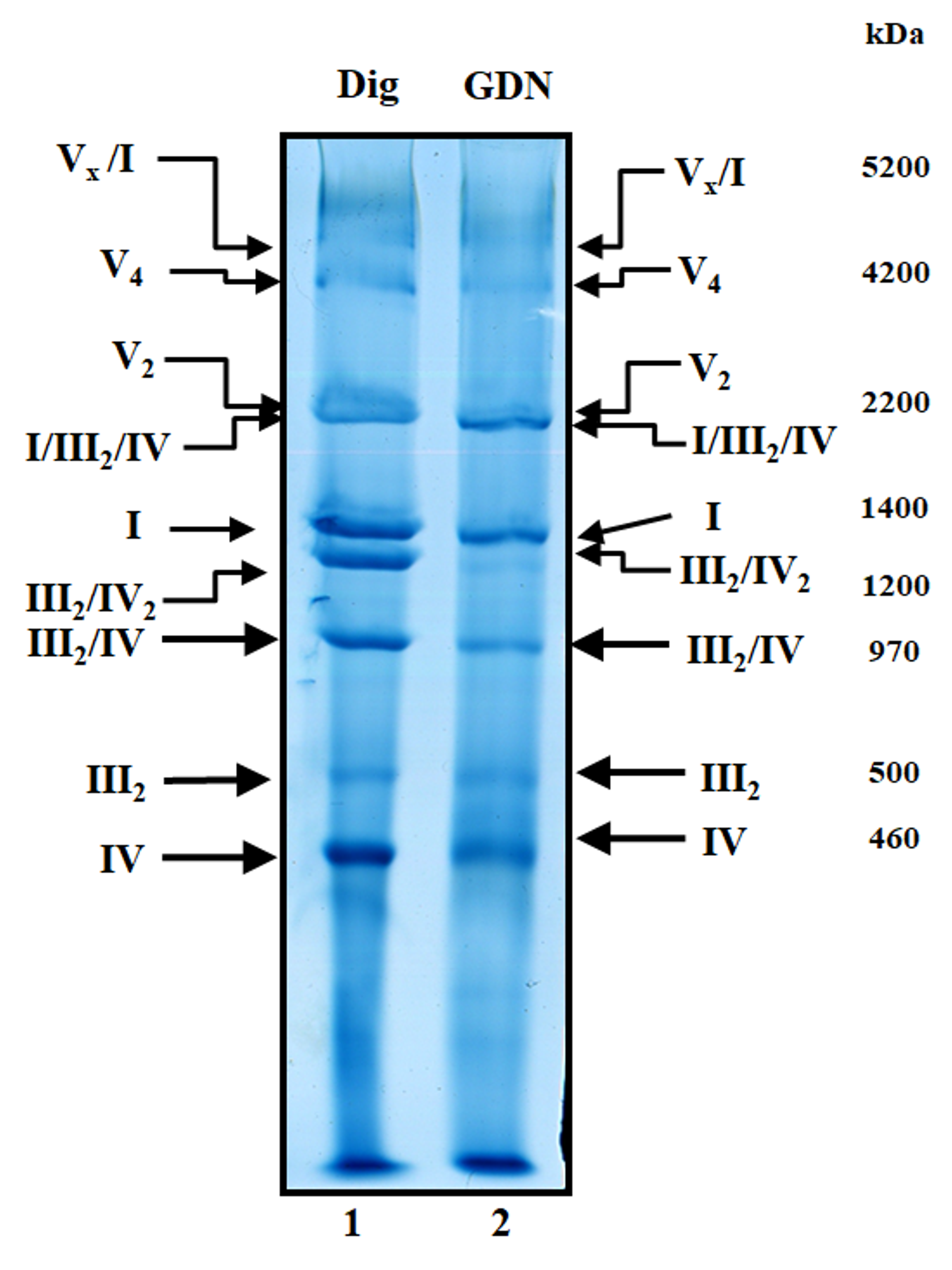

Supplement: Supplementary file 3 — ATPase oligomers and respiratory supercomplexes in Euglena gracilis extracted with non-ionic detergents. Isolated mitochondria were solubilized with digitonin (Dig) and its synthetic drop-in substitute glyco-diosgenin GDN101 (GDN) at 8.0 g/g protein. Final concentrations of detergent were 6.4%. After removing the insoluble material, each sample was resolved by BN-PAGE in a 3–10% polyacrylamide gradient gel. The determined molecular mass of each isolated complex or supercomplexes are indicated. Nomenclature used: I, III and IV for the corresponding mitochondrial complexes, V2 and V4 for the dimeric and tetrameric ATP synthase respectively. Supercomplexes were: III2/IV, III2/IV2, the so-called “Respirasome” association I/III2/IV and the putative Vx/I association, their stoichiometries are indicated as subindexes. (PNG 1146 kb) [file 10863_2021_9882_Fig8_ESM.png]

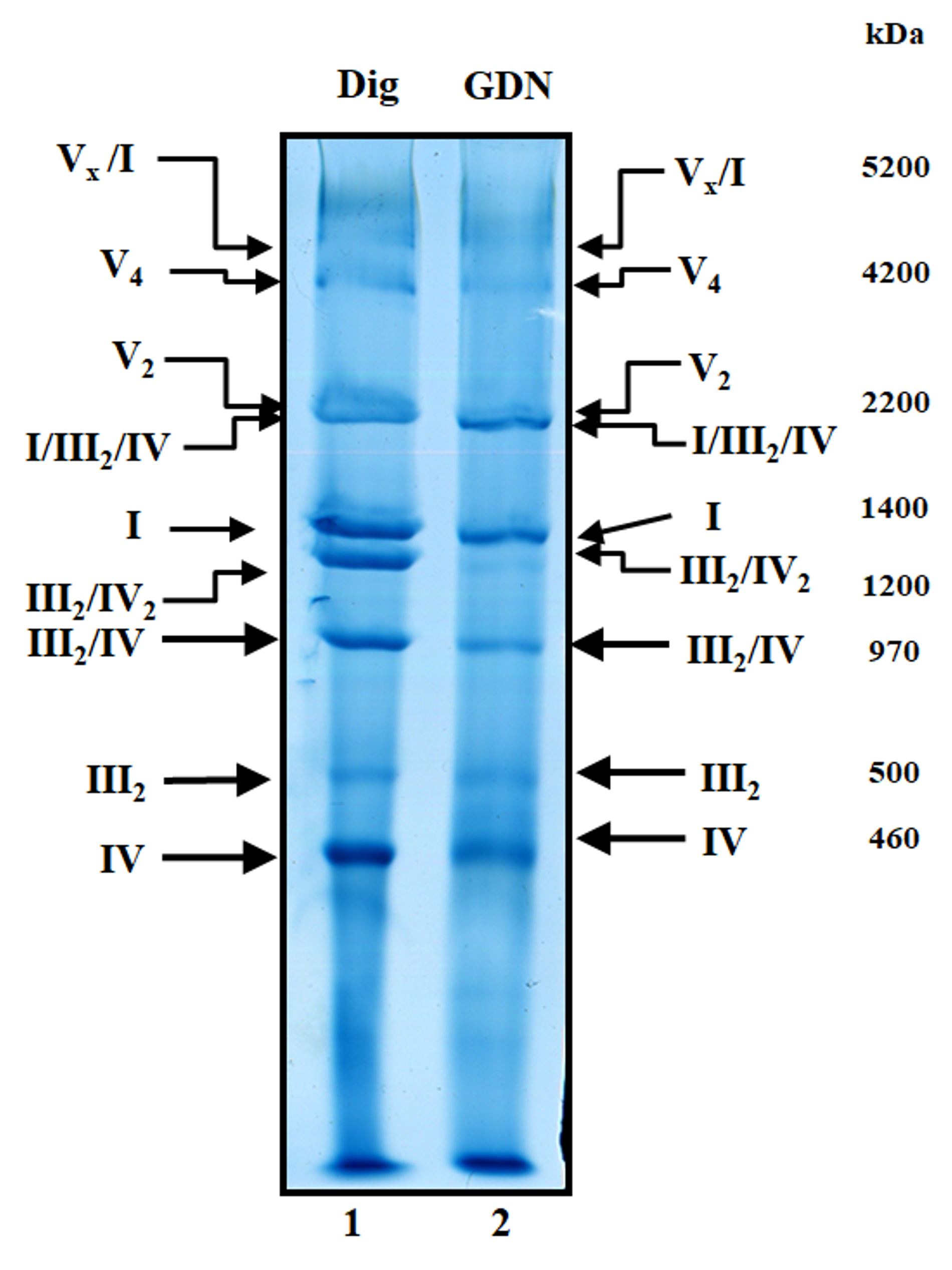

Supplement: Supplementary file 4 — High Resolution Image (TIF 18828 kb) [file 10863_2021_9882_MOESM2_ESM.tif]

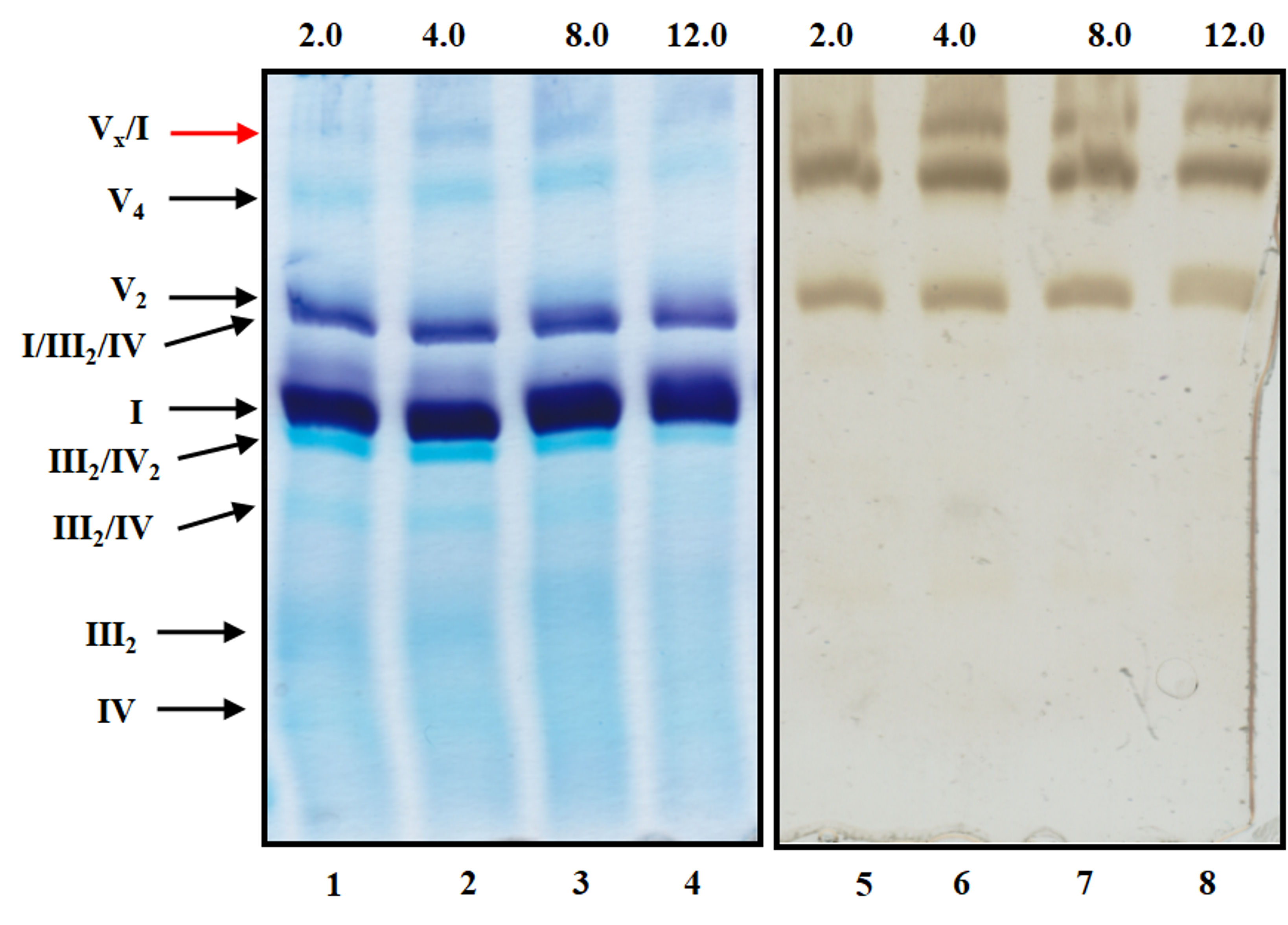

Supplement: Supplementary file 5 — Stability of the Euglena gracilis supercomplexes at increasing concentrations of digitonin. Isolated mitochondria were solubilized with increasing concentrations of digitonin. Left panel: in-gel NADH-dehydrogenase activity; the BN-gel was incubated in the presence of NADH and Nitro blue tetrazolium chloride (NBT). Right panel: BN-gel and detection of in-gel ATPase activity. The gel was incubated with ATP, MgSO4 and Pb(NO3)2. Numbers above represent the g of detergent/g of protein relation. (PNG 1880 kb) [file 10863_2021_9882_Fig9_ESM.png]

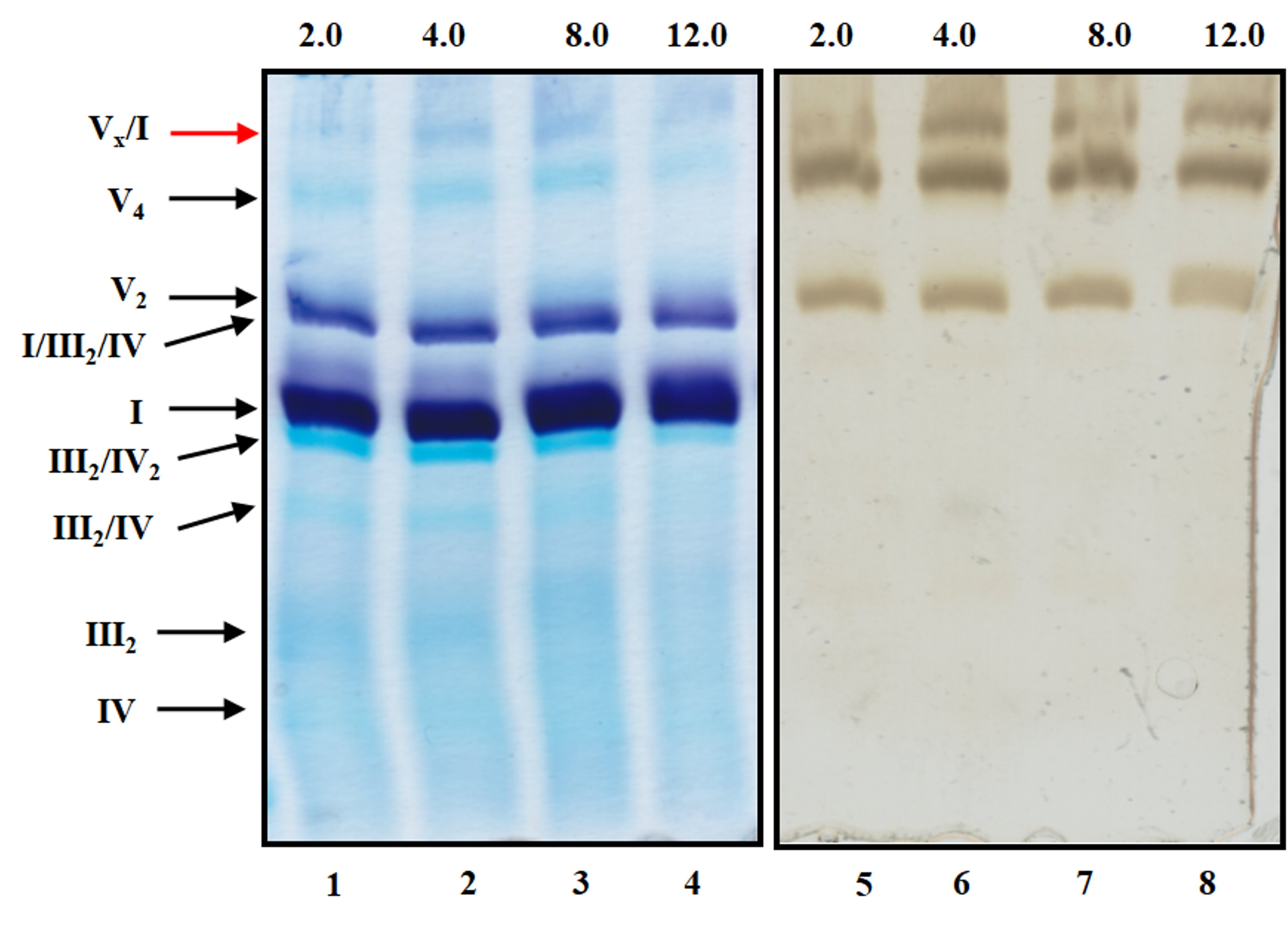

Supplement: Supplementary file 6 — High Resolution Image (TIF 30133 kb) [file 10863_2021_9882_MOESM3_ESM.tif]

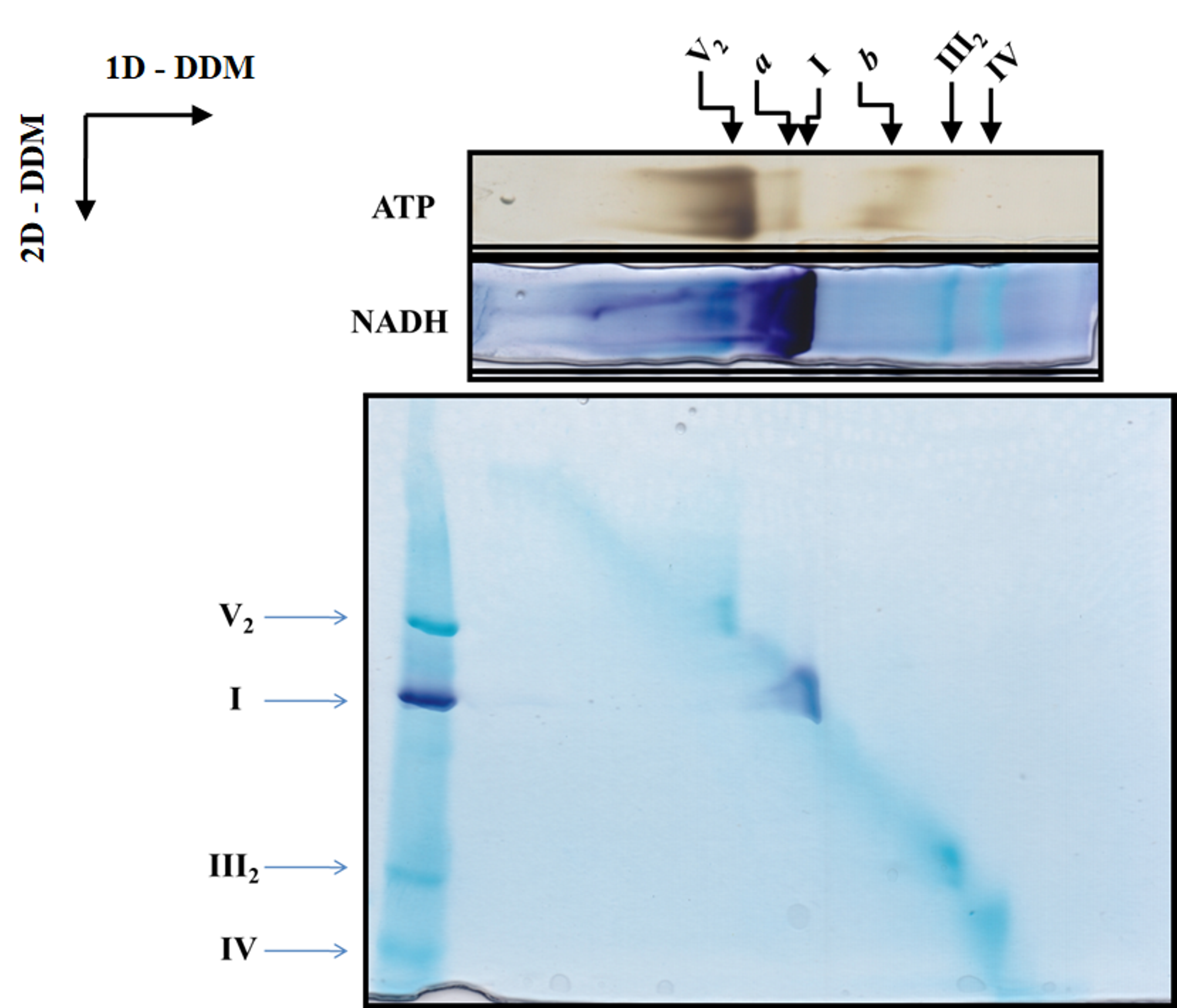

Supplement: Supplementary file 7 — Two-dimensional resolution of OXPHOS complexes in Euglena gracilis mitochondria. Upper lanes: The OXPHOS complexes from Euglena mitochondria were solubilized using n-dodecyl-β-D-maltoside (DDM) and separated by BN-PAGE followed by in-gel NADH-dehydrogenase and ATPase activities. Lower panel: NADH-dehydrogenase activity stain two-dimensional gels from DDM-extracted complexes, the isolated spots in the diagonal show the complexes separated by their molecular masses in each dimension. Isolated complexes were used as molecular mass markers (left lane). (PNG 1702 kb) [file 10863_2021_9882_Fig10_ESM.png]

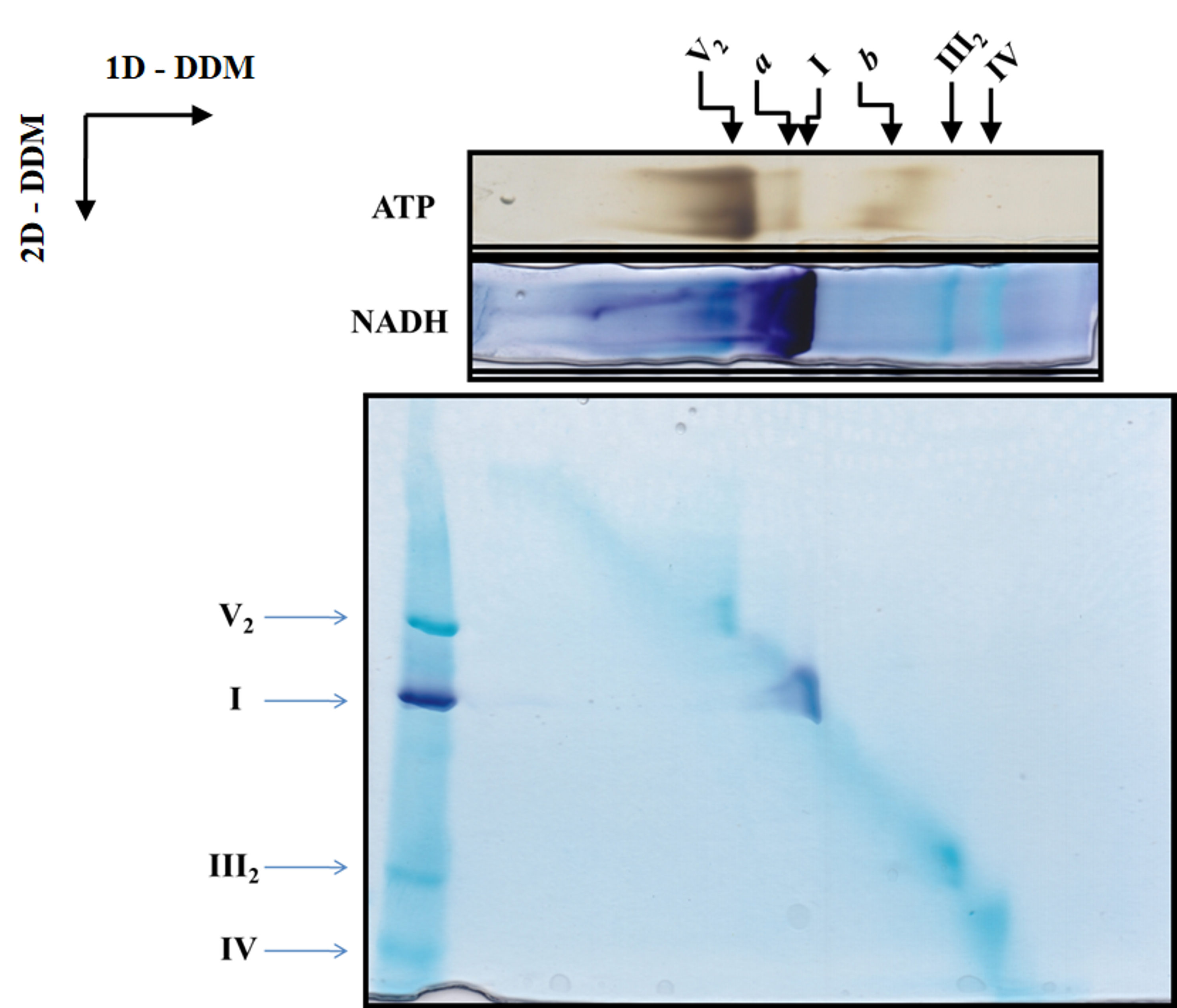

Supplement: Supplementary file 8 — High Resolution Image (TIF 29387 kb) [file 10863_2021_9882_MOESM4_ESM.tif]

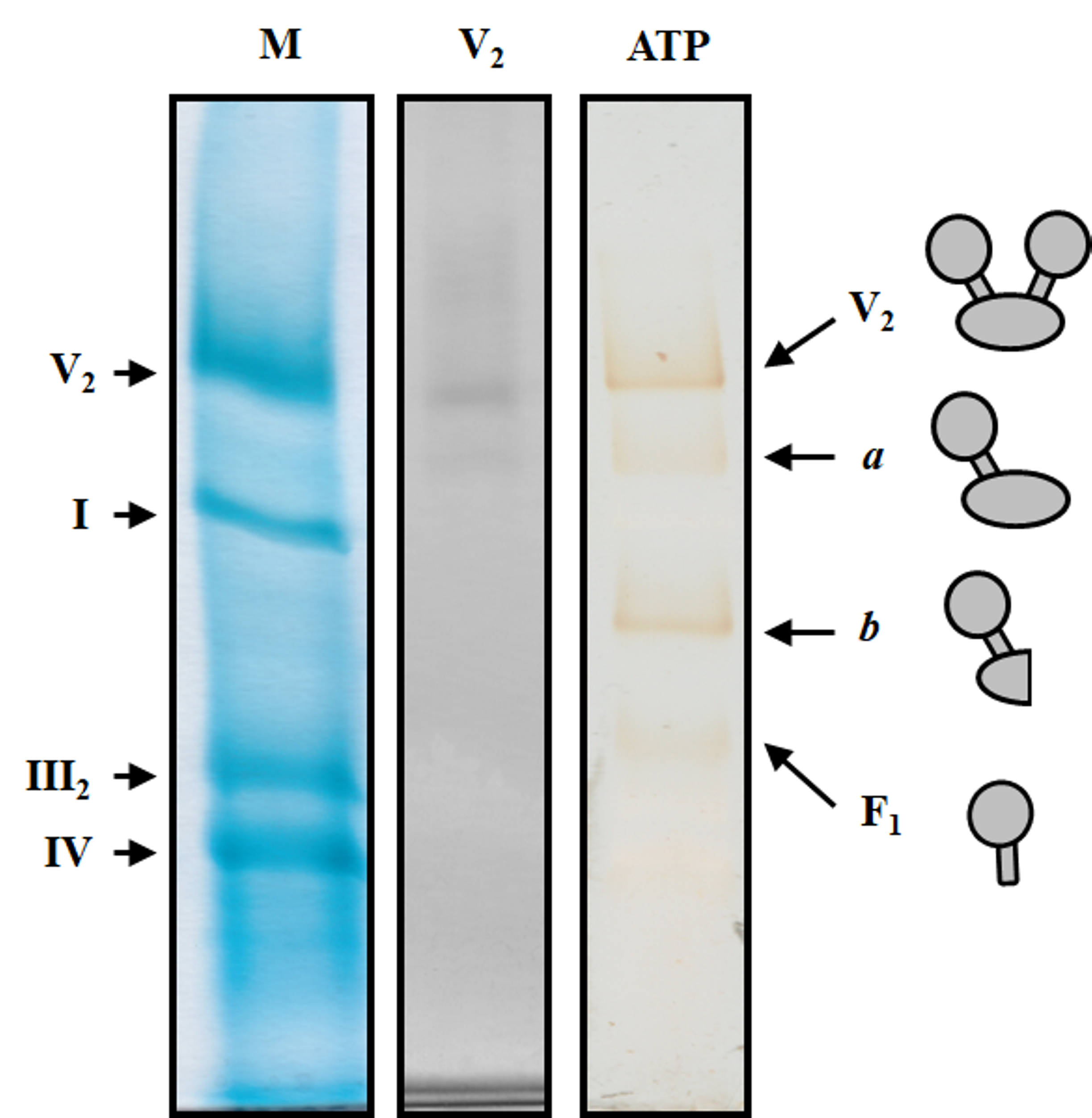

Supplement: Supplementary file 9 — ATP synthase dissociation in Euglena gracilis. Purified dimeric ATP synthase was incubated in presence of n-dodecyl-β-D-maltoside (DDM) at room temperature for 10 min. Four species are observed which may correspond to dimeric enzyme V2, dimer without one F1 sector (band a), monomeric enzyme (band b), and the F1 sector. This assignment was done based on size. (PNG 1164 kb) [file 10863_2021_9882_Fig11_ESM.png]

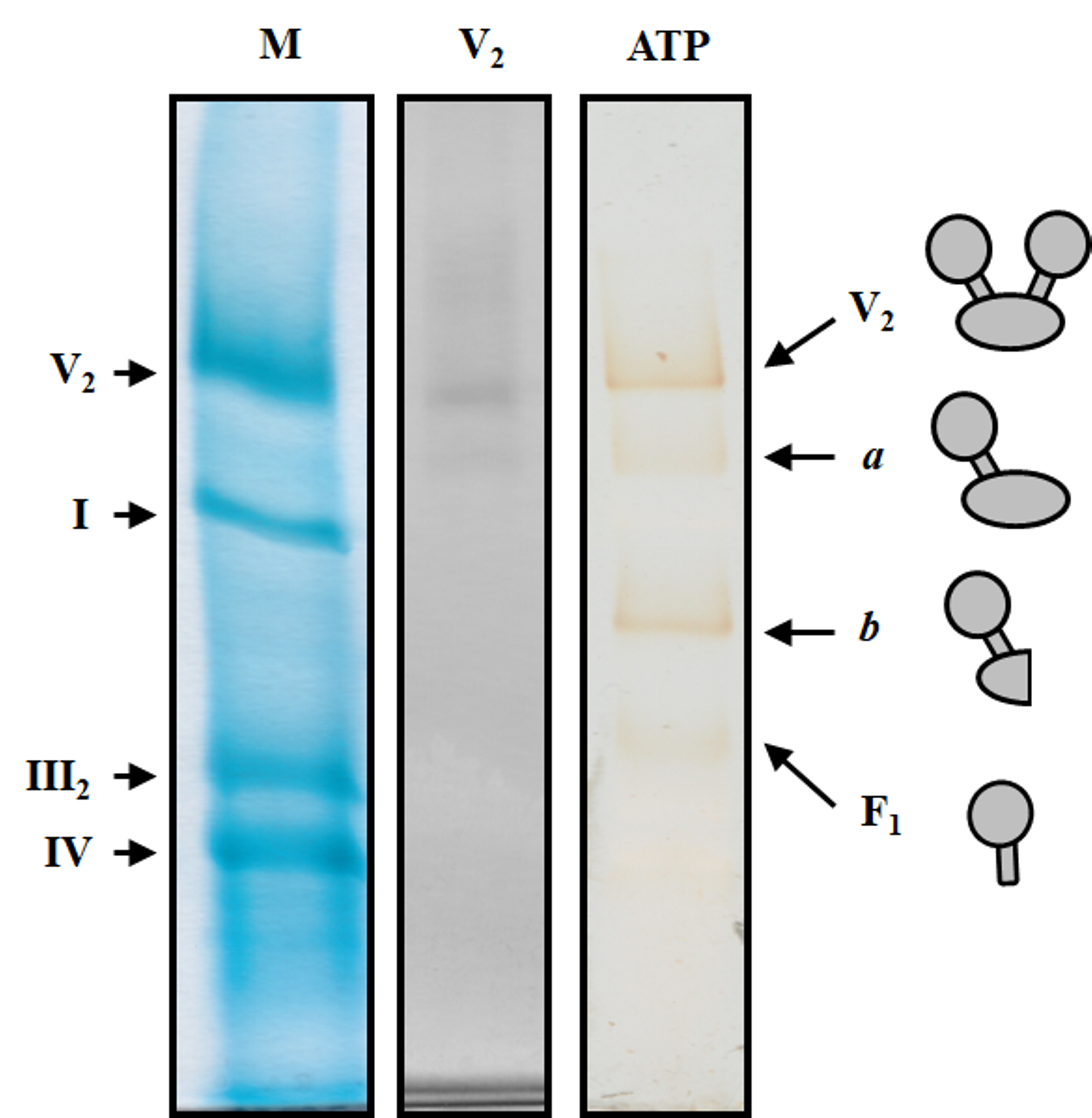

Supplement: Supplementary file 10 — High Resolution Image (TIF 23977 kb) [file 10863_2021_9882_MOESM5_ESM.tif]

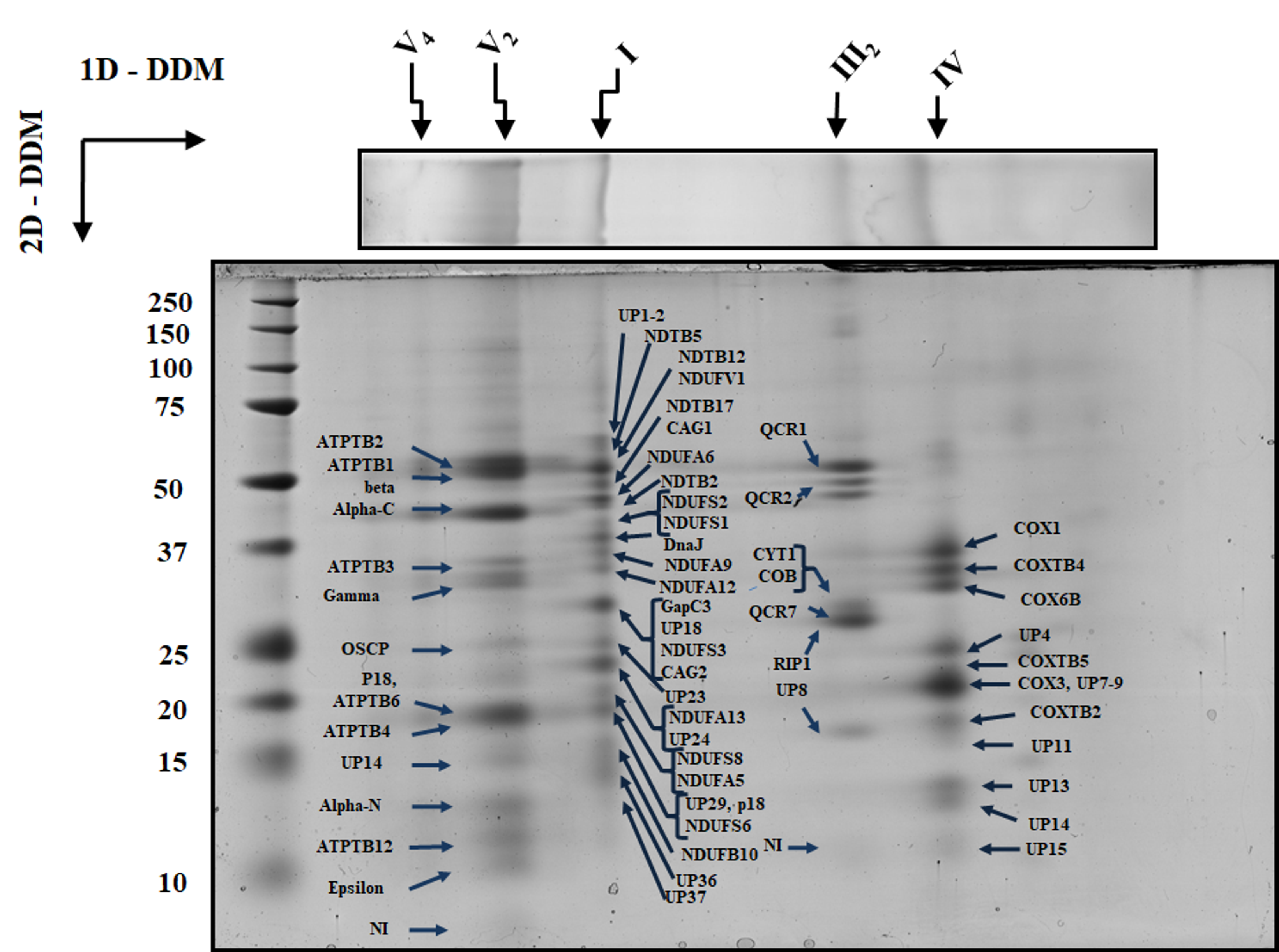

Supplement: Supplementary file 11 — Two-dimensional resolution of OXPHOS complexes in E. gracilis mitochondria. The OXPHOS complexes from Euglena mitochondria were solubilized using n-dodecyl-β-D-maltoside (DDM) (upper lane) and separated by BN-PAGE. Lower panel: Two-dimensional SDS-tricine gel from DDM-extracted complexes. Identified subunits by tandem mass spectroscopy of each complex are indicated (Yadav et al. 2017; Miranda-Astudillo et al. 2018b). Molecular masses from the molecular mass marker are indicated on left side. (PNG 2180 kb) [file 10863_2021_9882_Fig12_ESM.png]

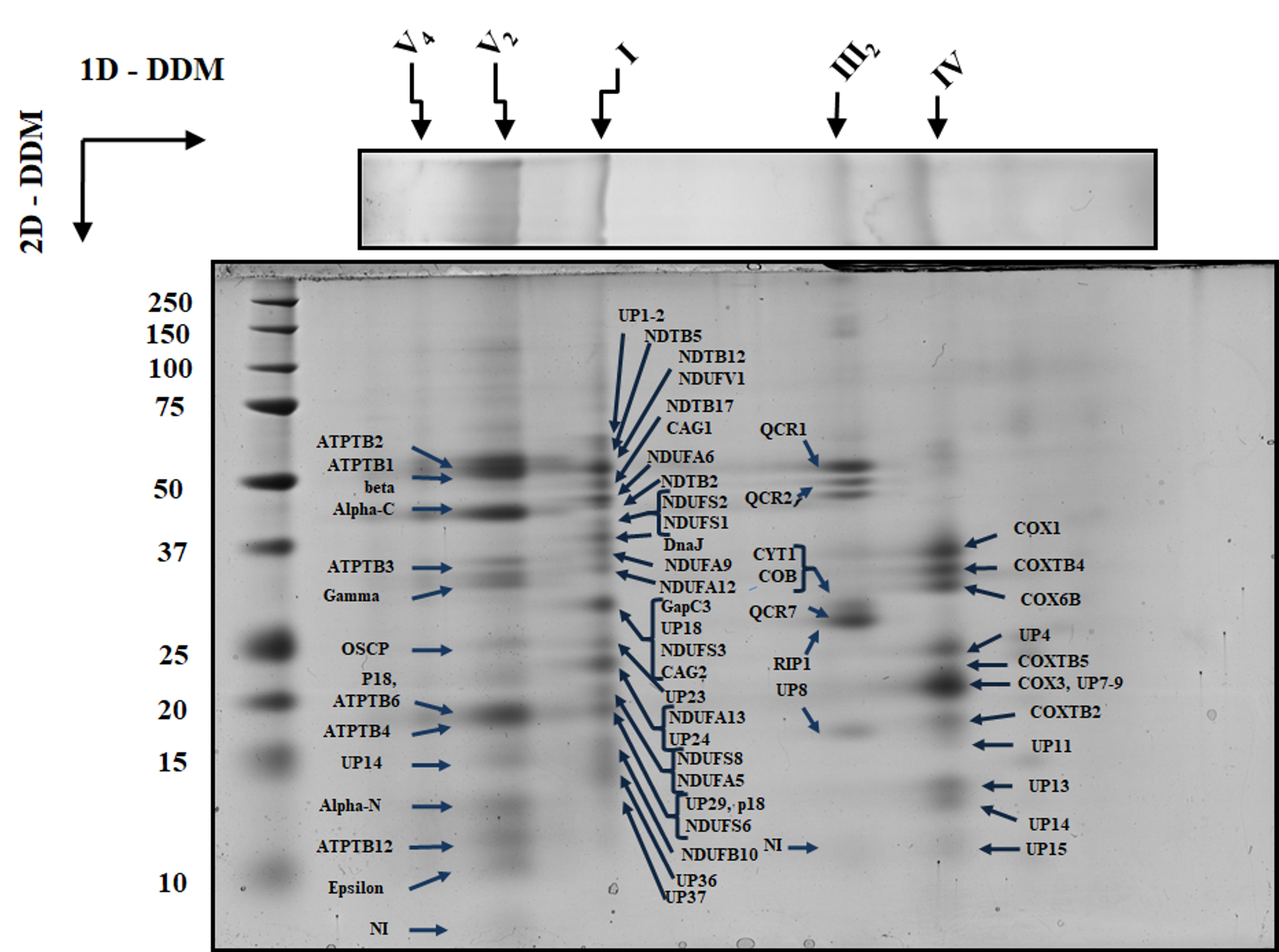

Supplement: Supplementary file 12 — High Resolution Image (TIF 31356 kb) [file 10863_2021_9882_MOESM6_ESM.tif]

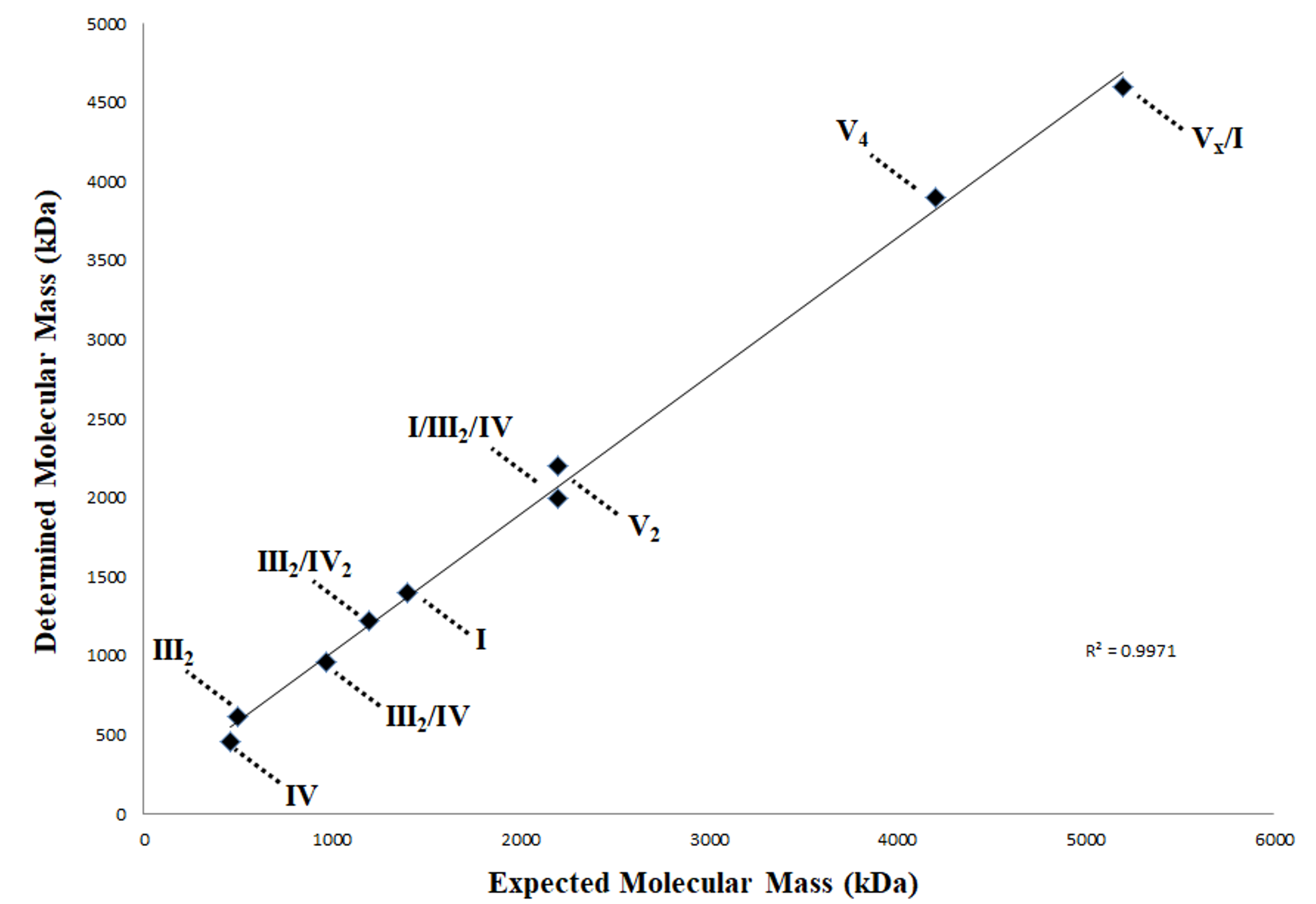

Supplement: Supplementary file 13 — Linear regression of calculated versus determined molecular masses in Euglena gracilis supercomplexes. (PNG 568 kb) [file 10863_2021_9882_Fig13_ESM.png]

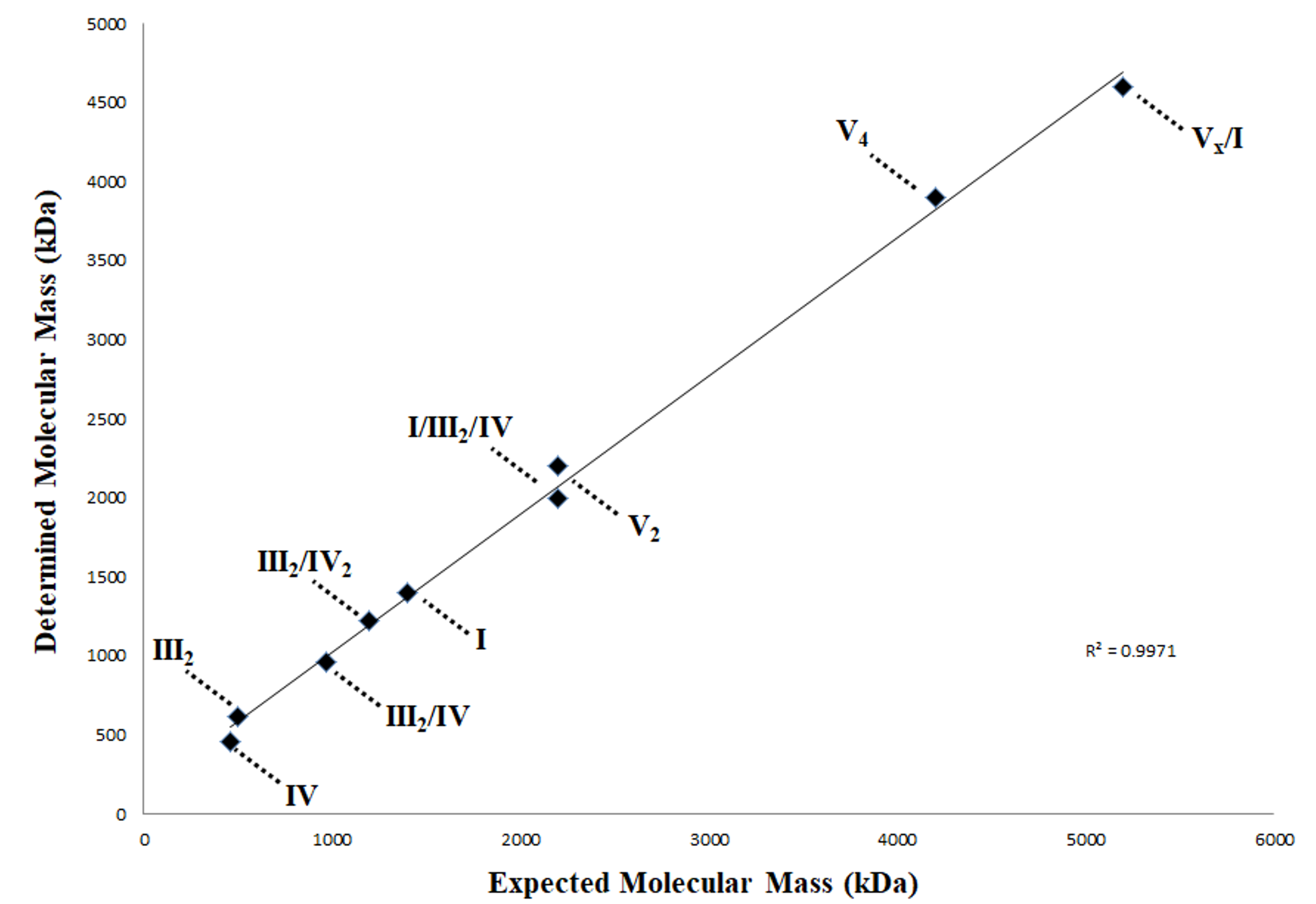

Supplement: Supplementary file 14 — High Resolution Image (TIF 28797 kb) [file 10863_2021_9882_MOESM7_ESM.tif]
